# Systematic identification of genetic influences on methylation across the human life course

**DOI:** 10.1186/s13059-016-0926-z

**Published:** 2016-03-31

**Authors:** Tom R. Gaunt, Hashem A. Shihab, Gibran Hemani, Josine L. Min, Geoff Woodward, Oliver Lyttleton, Jie Zheng, Aparna Duggirala, Wendy L. McArdle, Karen Ho, Susan M. Ring, David M. Evans, George Davey Smith, Caroline L. Relton

**Affiliations:** MRC Integrative Epidemiology Unit (IEU) & School of Social and Community Medicine, University of Bristol, Oakfield House, Oakfield Grove, Bristol BS8 2BN UK; Avon Longitudinal Study of Parents and Children (ALSPAC) & School of Social and Community Medicine, University of Bristol, Oakfield House, Oakfield Grove, Bristol BS8 2BN UK; University of Queensland Diamantina Institute, Translational Research Institute, Brisbane, 4102 Australia; Institute of Genetic Medicine, Newcastle University, Newcastle upon Tyne, NE1 7RU UK

**Keywords:** Methylation quantitative trait loci, mQTL, Cohort, Genetic association, DNA methylation

## Abstract

**Background:**

The influence of genetic variation on complex diseases is potentially mediated through a range of highly dynamic epigenetic processes exhibiting temporal variation during development and later life. Here we present a catalogue of the genetic influences on DNA methylation (methylation quantitative trait loci (mQTL)) at five different life stages in human blood: children at birth, childhood, adolescence and their mothers during pregnancy and middle age.

**Results:**

We show that genetic effects on methylation are highly stable across the life course and that developmental change in the genetic contribution to variation in methylation occurs primarily through increases in environmental or stochastic effects. Though we map a large proportion of the *cis*-acting genetic variation, a much larger component of genetic effects influencing methylation are acting in *trans*. However, only 7 % of discovered mQTL are *trans*-effects, suggesting that the *trans* component is highly polygenic. Finally, we estimate the contribution of mQTL to variation in complex traits and infer that methylation may have a causal role consistent with an infinitesimal model in which many methylation sites each have a small influence, amounting to a large overall contribution.

**Conclusions:**

DNA methylation contains a significant heritable component that remains consistent across the lifespan. Our results suggest that the genetic component of methylation may have a causal role in complex traits. The database of mQTL presented here provide a rich resource for those interested in investigating the role of methylation in disease.

**Electronic supplementary material:**

The online version of this article (doi:10.1186/s13059-016-0926-z) contains supplementary material, which is available to authorized users.

## Background

Epigenetic mechanisms play a central role in the regulation of cellular processes by influencing genomic activity [1]. DNA methylation, defined as the covalent bonding of a methyl group to a cytosine in the context of a CpG dinucleotide, is an important component of these mechanisms in mammals. Canonically, DNA methylation typically represses transcription, which can occur by inhibiting the binding of transcription factors or by recruiting DNA binding proteins that remodel chromatin structure. Consequently, the establishment and maintenance of DNA methylation patterns are crucial for normal cellular function and developmental processes and, indeed, these patterns are highly heterogeneous at different life stages [2] and between different tissue types [3].

Genome-wide DNA methylation can be considered to be a large set of measurable traits (one per CpG site) in which variation can arise from environmental [4], stochastic [5] or genetic [6] perturbations, and there is growing evidence that DNA methylation could mediate the relationship between these processes in influencing complex diseases [7]. An important step in understanding the processes underpinning DNA methylation is mapping the genetic factors that influence its variation. A recent study estimated that almost 20 % of reliably assayed variation in blood DNA methylation is heritable and that 50 % of CpG sites showed evidence of a significant genetic component [8]. A different perspective is provided by a study looking at the most variably methylated regions between neonates, which found 25 % were best explained by genotype alone and 75 % by a combination of genotype and environment [9]. Another large study showed that DNA methylation variation in adipose tissue was highly heritable (h^2^_median_ = 0.34) and that shared environmental effects correlated with metabolic phenotype-associated CpGs [10]. Heritability of CpG methylation levels in whole blood in young people has also been shown to correlate highly with stability of methylation at the same sites in later life [11]. However, those studies that have attempted to map genetic effects influencing DNA methylation (methylation quantitative trait loci (mQTL)) have so far only explained a small proportion of the genetic variance that is estimated to exist and it is evident that much larger sample sizes will be required to map the majority of the predicted genetic effects.

One can address the question of how genetic variation influences DNA methylation in a number of ways and here we focus on three specific research avenues. First, the genetic architecture of methylation variation can provide information about the level of complexity that underlies population-level differences. This can be examined by (a) estimating the proportion of explanatory common genetic variation (mQTL) that occurs close to the methylation site (in *cis*) versus the proportion that occurs elsewhere in the genome (in *trans*), and (b) the level of polygenicity of the genetic component for *cis* and *trans* regions.

Second, in characterising and mapping mQTL it is of interest to know the extent to which genetic effects are stable over time. Because epigenetic change is a cornerstone of mammalian development, elucidating whether genetic effects have a consistent influence across the life course or are specific to certain developmental windows is important for gauging the extent to which mQTL could be involved in epigenetic restructuring and perturbation of developmental trajectories.

Third, a comprehensive catalogue of mQTL can be used to investigate (a) whether those regions of methylation that are influenced by genetic variation are likely to be inert or are involved in cellular function and (b) if these elements are functional, then to what extent do mQTL influence complex disease as a consequence of their influence on DNA methylation.

Here we present a comprehensive genome-wide *cis* and *trans* mQTL longitudinal analysis in blood DNA at three time points in the life course of a large number of participants in the Avon Longitudinal Study of Parents and Children (ALSPAC) [12] and two time points in the life course of their mothers [13], in the form of an online searchable database (http://www.mqtldb.org/). We assess the stability of mQTL across the life course and identify the biological pathways in which they function. We evaluate the relationship of mQTL with other downstream phenotypes, including gene expression, traits and diseases, and quantify the contribution made by mQTL to genetic variance in several common complex diseases that have previously been the subject of genome-wide association studies (GWAS).

## Results

### *Cis* and *trans* mQTL mapping

The ARIES dataset [14] represents DNA methylation levels collected at five different time points across the life course from individuals in ALSPAC: in young people we collected samples at birth (cord blood, n = 771), childhood (n = 834) and adolescence (n = 837); in their mothers we sampled during pregnancy (n = 764) and in middle age (n = 742) (Additional file 1: Table S1). We performed an exhaustive whole-genome mQTL analysis by testing approximately 8.3 million common single-nucleotide polymorphisms (SNPs) against each reliable CpG probe (395,625 out of 485,577) in each time point (Additional file 1: Table S2). After conservative multiple testing correction (*p* < 1 × 10^−14^) we identified between 24,262 and 31,729 sentinel associations at each time point (Table 1; Additional file 1: Table S3). Approximately 93 % of the mQTL were acting in *cis* (defined as within ±1 Mb of the CpG probe on the basis of previous a previous report [15] and our own observation of the distribution of SNP/CpG distances, although definitions of *cis* in the literature vary widely from a few hundred base pairs [16] to 1 Mb [17, 18]).

**Table 1:** Number of mQTL and associated CpGs reaching the significance threshold for each time point

Counts	Birth	Childhood	Adolescence	Pregnancy	Middle age
Sentinel mQTL					
*Cis* ^a^	24,262	31,729	30,294	29,038	27,043
*Trans* ^b^	1979	2658	2442	2394	2144
Conditionally independent^c^	2705	5446	5040	4454	3463
Total mQTL	28,946	39,833	37,776	35,886	32,650
Total unique CpGs	27,387	36,705	34,886	33,344	30,676

^a^Number of CpG sites with a *cis* SNP

^b^Number of independent (±1 Mb) *trans* effects

^c^Number of mQTL further detected after performing conditional analysis

**Fig. 1:**
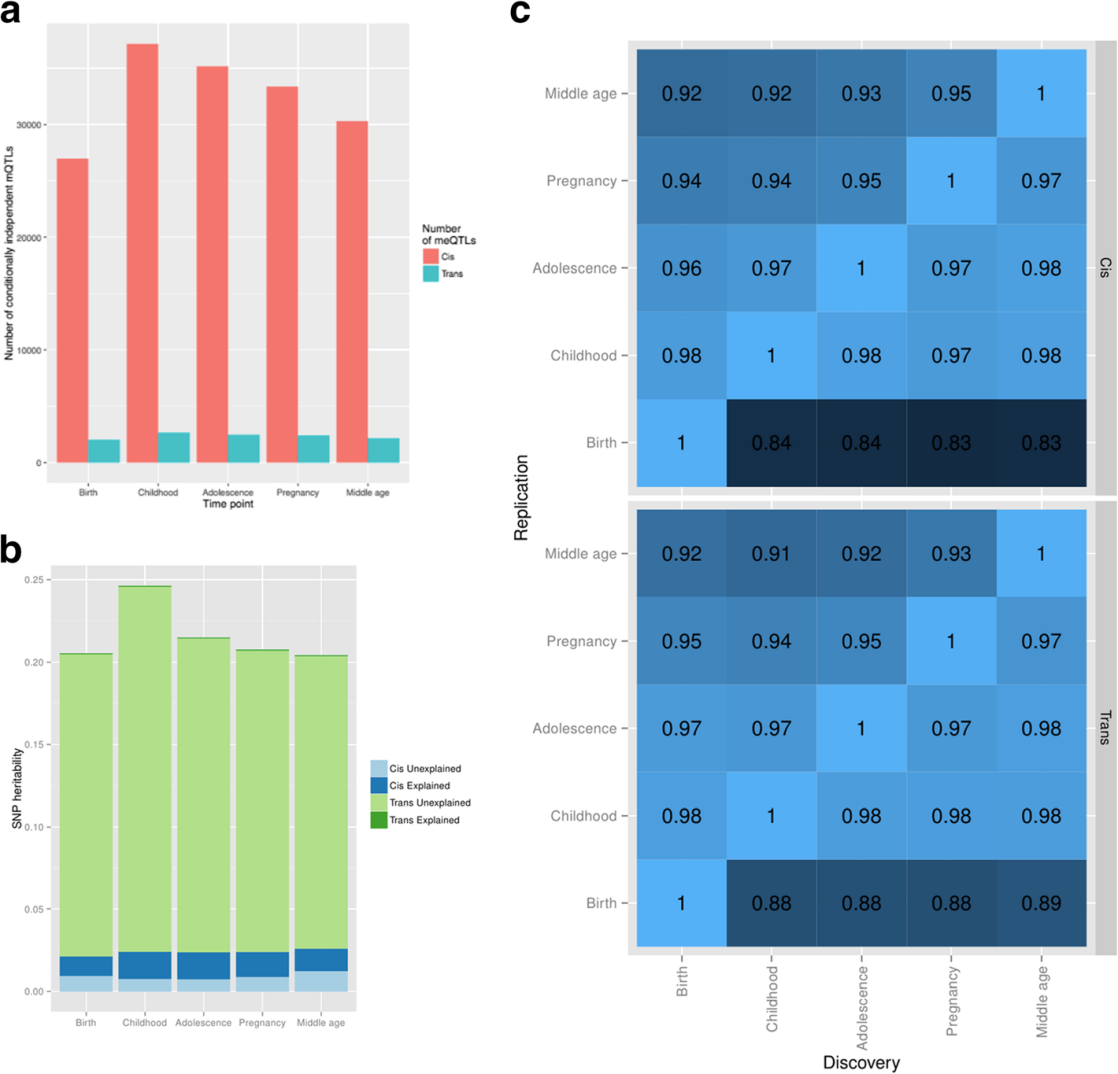
Temporal pattern of mQTL. **a** The total number of *cis* and *trans* mQTL discovered at each time point. **b** Total bars represent the SNP heritability at each time point. Each bar is split into genetic variation due to common SNPs acting in *cis* (*blue*) and *trans* (*green*). *Cis* and *trans* variation is further divided into the proportion that is explained by mapped SNPs (*p* < 1 × 10^−14^). **c** The proportion of discovered mQTL at a specific time point that replicate at *p* < 1 × 10^−7^ in each of the other time points. *Darker colours* correspond to lower replication rates

We also performed conditional analysis which identified between 2705 and 5446 further mQTL at each time point that showed secondary, tertiary and quaternary effects also acting in *cis* (Additional file 1: Figure S1), giving 28,946–39,833 mQTL discovered at each time point influencing a total of 43,897 CpG sites across the genome (Table 1, Fig. 1a). The effect sizes as difference in median proportion methylated between homozygote groups is presented in Additional file 1: Figure S2.

### Genetic architecture of methylation variation

DNA methylation may be influenced by both genetic and environmental factors. To address the question of the relative contribution of genetic variation we used genomic restricted maximum likelihood (GREML) [19]. Here we estimated how much of the total variation in each methylation probe was captured by all 1.1 million common HapMap3 [20] tagging SNPs (minor allele frequency (MAF) > 0.01) to estimate what is known as the SNP heritability. Although the standard error is high for any one probe, when performed on all reliable probes at five time points this analysis enabled us to estimate the distribution of genetic contribution to methylation variation (SNP heritability) and how it varies over time. In addition, for each probe we partitioned the genetic variance into two components, the first using *cis* SNPs only and the second using *trans* SNPs only.

The results demonstrate that although the majority of mQTL act in *cis*, the majority of estimated genetic variation that influences methylation levels is acting in *trans* (Fig. 1b). This applies even if we extend the definition of *cis* to include the entire chromosome (data not shown).

### Variance explained by detected mQTL

Having partitioned the phenotypic variance of each CpG probe into *cis*-acting genetic variance, *trans*-acting genetic variance and environmental variance (which includes any genetic variance not captured by 1.1 million HapMap3 SNPs), we then went on to estimate how much of the genetic variation was ‘explained’ by the mQTL that we had detected in our association analyses. We estimated that the discovery mQTL detected in our association analysis explained over half of the proportion of total *cis* variation, whereas the *trans* mQTL explained less than 1 % of total *trans* variation. This result is consistent with the hypothesis that genetic perturbation close to the CpG site tends to have large effects whereas the complex network of regulatory interactions and a large mutational target size makes the *trans* component, on average, highly polygenic.

### Stability of genetic effects over time

DNA methylation changes in response to environmental exposures. We addressed the question of how stable the mQTL effects were over time by estimating the rate of replication of discovery mQTL from one time point in all other time points. Fig. 1c and Additional file 1: Figure S3 show that the proportion that replicated at a threshold of *p* < 1 × 10^−7^ was typically in excess of 95 %, although the rate of replication of post-birth discovery mQTL in the birth time point was consistently lower (84–86 %).

The distributions of estimated SNP heritabilities illustrate that average SNP heritability gradually falls from 0.24 in childhood to 0.21 at middle age (Fig. 1a, b). A regression of SNP heritability on age indicated a reduction of heritability of −0.0009 per year from childhood to adulthood (–0.0009, se=1.6e–5). There are two simple explanations for this observation: first, that the influence of genetic variation is reducing over time; or second, that the influence of environmental or stochastic perturbations is increasing over time. In the former case we would expect that the average coefficient of variation of methylation to decrease over time due to there being fewer genetic factors, whereas in the latter case we would expect it to increase due to more environmental factors or higher stochasticity. The latter explanation is supported by a clear increase (3.0 %, standard error 0.015 %) in the average coefficient of variation of methylation from childhood to middle age (data not shown).

In addition, while replication of mQTL from middle age to childhood is high, replication of mQTL from childhood is lower in later time points (Fig. 1c). Together this suggests that genetic effects are largely stable and that environmental or stochastic perturbation is gradually increasing over the life course, leading to lower SNP heritability estimates and lower power to detect mQTL as age increases. Two caveats to these observations are that the later two time points are different individuals from the earlier ones and they are comprised exclusively of women.

The stability of genetic effects is surprisingly high given the observational correlations of DNA methylation between different time points (Additional file 1: Figure S4). However, we observe that the mean correlation of methylation probes that have at least one significant mQTL is substantially higher than the average value (e.g. for all probes $\overline{r}=0.09$ and for mQTL probes $\overline{r}=0.31$ when comparing childhood with adolescence; Additional file 1: Figure S5).

### Long range influences of methylation levels

In contrast to what has been seen in expression quantitative trait loci (eQTL) studies, there is little evidence of individual mQTL influencing many CpG sites across the genome, with the vast majority of *trans* mQTL in our data just representing a single or small number of associations (Additional file 1: Figure S6). To gauge the extent to which methylation levels were influenced by CpG sites elsewhere in the genome, we performed a mediation analysis testing for mediation of *trans* mQTL effects by *cis* methylation. Mediation analyses are particularly susceptible to measurement error in the mediator (which will attenuate estimates of mediation). However, we provide some evidence that, amongst mQTL with both *cis* and *trans* effects, a proportion demonstrate some degree of mediation of the *trans* association by *cis* CpG sites. Additional file 1: Figure S7 presents this mediation analysis comparing a regression of *trans* CpG on SNP to a regression of *trans* CpG on (SNP + *cis* CpG). Whilst a large number of sites follow the y = x diagonal (providing little evidence of mediation), a proportion deviate from this line, showing that if the effects of *cis* methylation are taken into account, the *trans* association is attenuated. This non-independence between *cis* and *trans* effects at these loci does not prove a causal effect of *cis* methylation on *trans* methylation. To determine the likely impact of measurement error on this analysis, we present simulations of the effect of measurement error in the *cis* methylation variable in Additional file 1: Figure S8, which illustrates that our observed results are likely to underestimate the true extent of potential mediation in the presence of measurement error.

### Functional annotation

If mQTL are functionally important, they are likely to be distributed differentially across genomic features. To address this question, we analysed both mQTL and mQTL-associated CpG sites to determine their distribution across genomic regions. The distribution of DNA methylation at mQTL-associated CpGs in different contexts (genic features, CpG islands, shelves and shores) is illustrated in Additional file 1: Figures S9 and S10. This illustrates consistency in distributions between time points, but with notable differences between locations (consistent with observations by Shi et al. [21]).

**Fig. 2:**
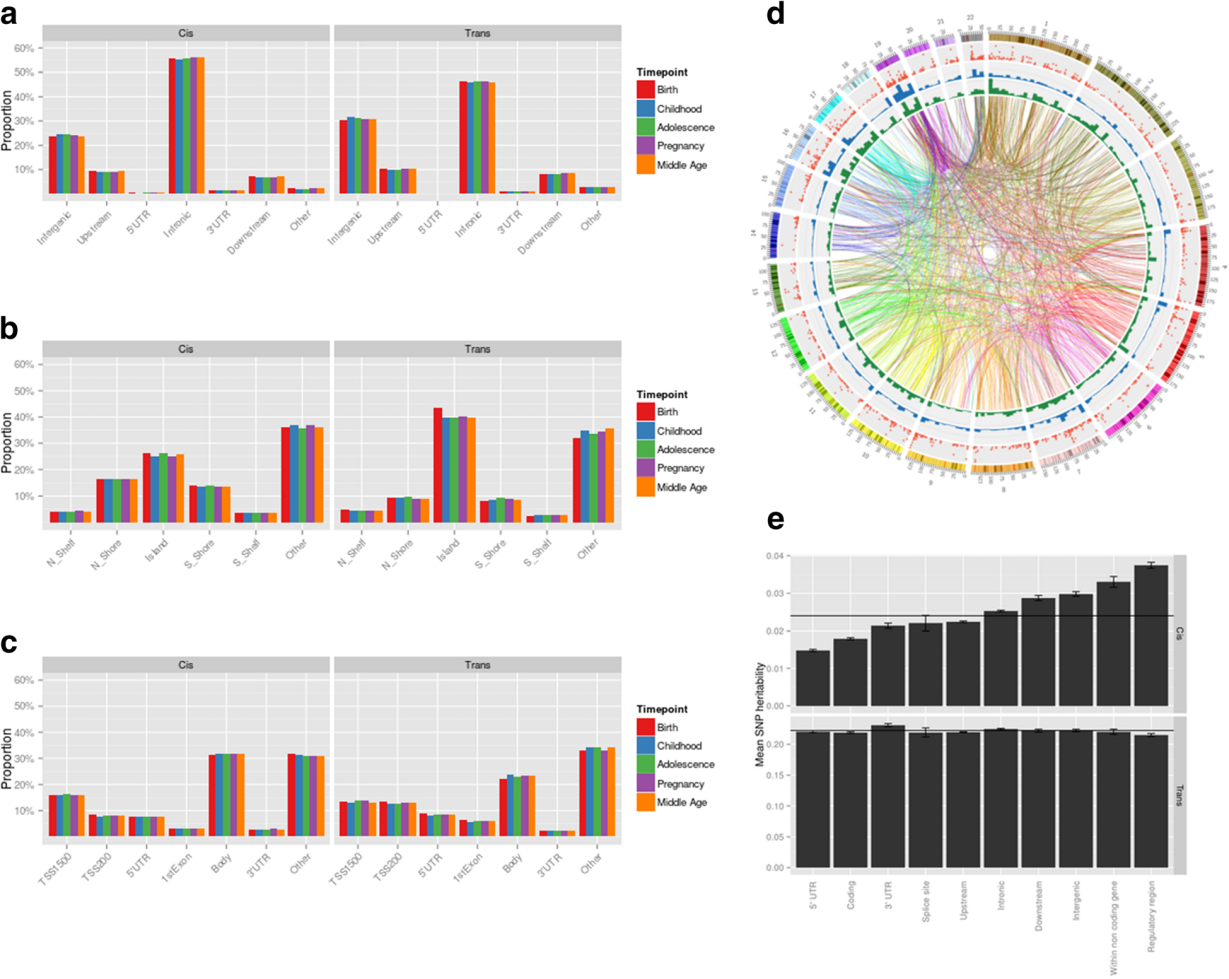
Genomic distribution of mQTL. **a** Distribution of mQTL across genomic features; **b** distribution of mQTL-associated CpG sites across CpG islands; **c** distribution of mQTL-associated CpG sites across genic features. **d** Circos plot illustrating *trans* mQTL at birth (see Additional file 1: Figure S5 for other time points). From the outside: chromosomes, −log_10_(*p* value) for association (*red points*), density of mQTL (*blue bars*), density of associated CpGs (*green bars*), density of genes (*gray bars*), *trans* associations between SNP and CpG (*lines*). **e** Average estimated *cis* (*top*) and *trans* (*bottom*) SNP heritability for methylation levels at different genomic features. Bar heights show mean heritability for each genomic feature. Error bars show standard error of the mean heritability. Horizontal lines indicate the mean heritability across all features. *UTR* untranslated region

The distribution of *cis* and *trans* mQTL across genic features at each time point (adjusted for overall representation within each feature) is presented in Fig. 2a–c. More than 65 % of *trans* associations were trans-chromosomal. Higher densities of *trans*-associated SNPs appear to occur on chromosomes 16, 17 and 19, illustrated in the circos bar charts in Fig. 2d showing density of associated CpGs and SNPs, but these do correspond closely with regions of higher gene density. The SNP heritability estimated by common *trans* SNPs was, on average, consistent for methylation levels at different genomic features, but there was a substantial relative increase of *cis* genetic influence on methylation levels at regulatory regions compared with coding regions (Fig. 2e).

Regional association plots for the top 25 *cis* associations at each time point are presented in Additional file 1: Figure S11, demonstrating a range of different patterns of methylation depending on linkage disequilibrium and correlation of methylation across the surrounding region. There is little evidence of individual mQTL influencing many CpG sites across the genome, with the vast majority of *trans* mQTL in our data just representing a single or small number of associations. However, this is very likely to reflect a lack of power to detect effects in the current sample size.

Overlap between our mQTL and eQTL reported previously by the GTEx consortium [22] is illustrated in Additional file 1: Figure S12. Amongst *cis* QTL more loci are both eQTL and mQTL than mQTL only. In a gene ontology comparison of our mQTL with the eQTL reported by Westra et al. [23], enrichment for genes from all mQTL results showed little evidence of informative enrichment (Additional file 1: Figure S13 and S14), but we found that *cis* mQTL also reported as eQTL are enriched for tissue development terms, whilst *trans* mQTL reported as eQTL are enriched for terms related to plasma membrane and cell periphery (Additional file 1: Figure S15).

### Influence of mQTL on disease

**Fig. 3:**
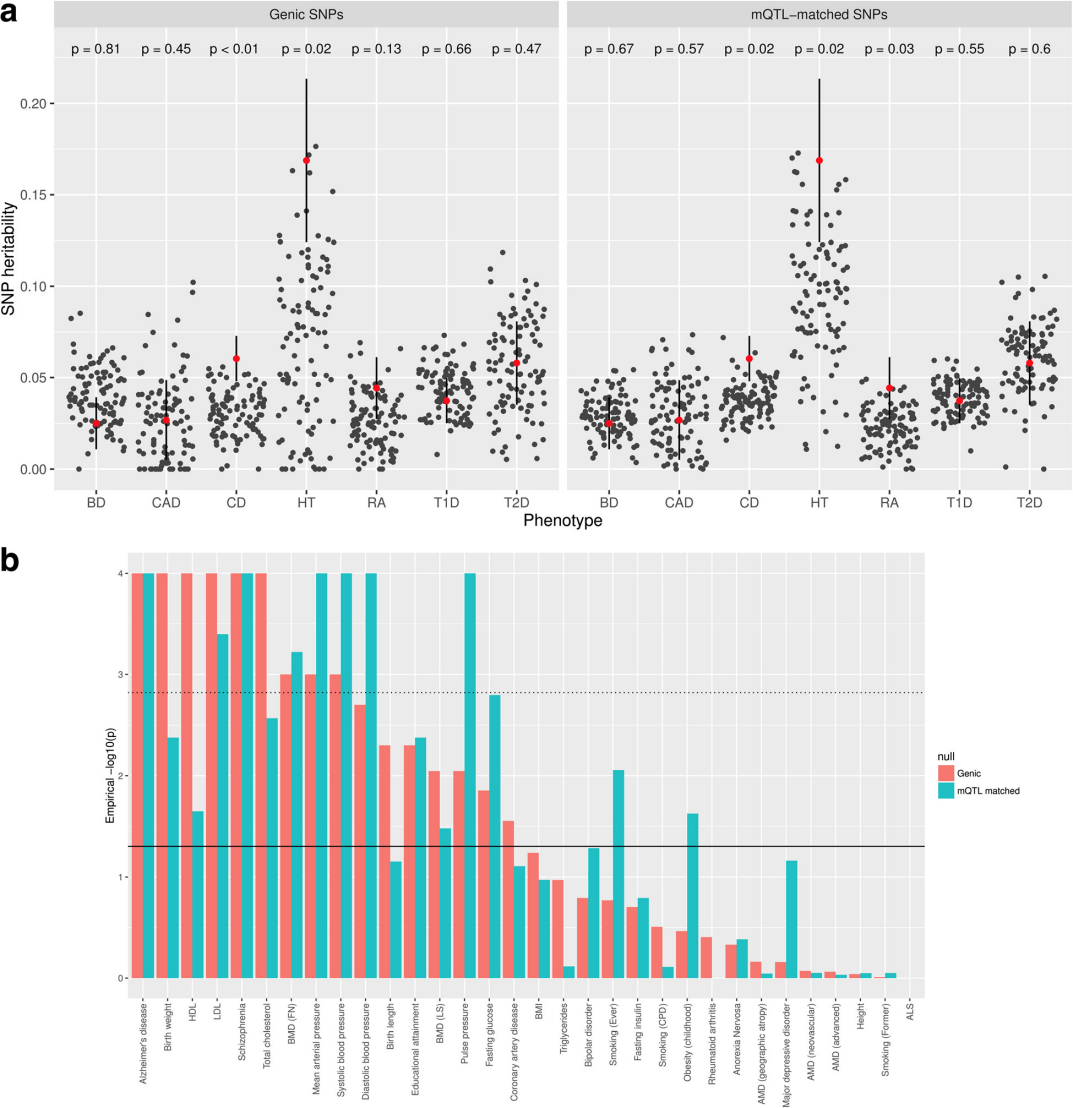
mQTL enrichment in diseases and traits. **a** Contribution of mQTL identified at each time point to variance of WTCCC common diseases bipolar disorder (*BD*), coronary artery disease (*CAD*), Crohns disease (*CD*), hypertension (*HT*), rheumatoid arthritis (*RA*), type 1 diabetes (*T1D*) and type 2 diabetes (*T2D*). *Red dots* represent the component of a trait’s genetic variance attributable to *cis*-acting mQTL SNPs with significance levels of *p* < 1 × 10^−14^, on the liability scale, excluding chromosome 6. *Black points* depict the point estimates of SNP heritability estimates under the null hypotheses of SNPs coming from genic regions (*left plot*) or SNPs with the same proportion of genic features as the mQTLs (*right plot*). *P* values relate to the proportion of the null estimates that surpass the mQTL estimates. **b** Enrichment analysis of *cis*-acting mQTL SNPs with significance levels of *p* < 1 × 10^−14^ in large-scale GWAS summary statistics for 33 complex traits. The *solid horizontal line* denotes empirical *p* value of 0.05 and the *dotted line* shows the threshold after correcting for multiple testing. *Red bars* are based on a null of genic SNPs, *blue bars* on a null of mQTL-matched SNPs. HDL = high-density lipoprotein cholesterol, LDL = low-density lipoprotein cholesterol, BMD = bone mineral density, FN = femoral neck, LS = lumbar spine, BMI = body mass index, AMD = age-related macular degeneration and ALS = amyotrophic lateral sclerosis

Though it is clear that the genetic component of methylation variation is substantial, the broad influence of variation in methylation on distal traits and disease outcomes is yet to be established. Observational associations between methylation and outcome are insufficient to ascertain causality. An alternative approach is to test if the *cis* mQTL that are likely to have direct effects on methylation levels also influence complex traits. To test this we partitioned the SNP heritability of seven complex traits in the Wellcome Trust Case Control Consortium (WTCCC) [24] data into a component for *cis* mQTL and a component for all HapMap3 [20] tagging SNPs. For comparison we compared our results against two different null hypotheses: is the variance explained by the mQTLs more than would be expected by chance (a) from the same number of SNPs sampled from genic regions and (b) if we sample the same number of SNPs to have the same distribution of genomic annotations as the mQTLs (Fig. 3a; Additional file 1: Figure S16)? In both cases we match our null SNPs to our mQTLs on allele frequency and linkage disequilibrium (LD) structure also.

Our results suggest that, for Crohn's disease, hypertension and rheumatoid arthritis, the proportion of SNP heritability attributed to mQTL was greater than expected under the null hypothesis that their contribution was solely due to residing within a particular set of genomic annotations (null hypothesis ‘b’). However, note that the evidence for these results is weak (*p* > 0.05) if multiple testing is taken into account using a Bonferroni correction. An additional limitation of this analysis is that the WTCCC controls (used for all seven diseases) were normal population samples and, therefore, may have included some cases. We next used our discovery mQTL to test for enrichment of low *p* values in publicly available GWAS results from large meta-analyses (Fig. 3b). We observed evidence that mQTL were more enriched for low *p* values in GWAS results than (a) randomly sampled genic SNPs and (b) mQTL annotation-matched SNPs for 9 and 8 out of 33 complex traits (Bonferroni corrected *p* < 0.05/33), respectively. Those complex traits showing enrichment for *cis* mQTL in GWAS results against both null hypotheses were Alzheimer’s disease, schizophrenia, low-density lipoprotein cholesterol, bone mineral density and blood pressure. These results suggest that there may be a genetic influence via methylation on downstream complex traits and that it is likely that very many CpG sites contribute to complex traits, each contributing only a small effect.

## Discussion

We present a large-scale analysis of blood mQTL in children and their mothers at multiple time points through the life course. We found that, whilst methylation itself is not highly stable over time, the effects of mQTL are surprisingly consistent, both within the same individuals and across a generation. This stability is consistent with another study that found significant overlap in mQTL between developmental stages (in addition to between ancestral groups and tissue types) [9]. Average SNP heritability of DNA methylation exceeded 0.2 at all time points, with *trans* effects estimated to account for the majority of this heritability. This is consistent with previously reported total heritability on the Illumina Infinium HM450 array of 0.187 [8]. However, the proportion of *trans* heritability explained by our *trans* mQTL is much smaller than the proportion of *cis* heritability explained by our *cis* mQTL. This is most likely due to polygenic *trans* effects, with many *trans* mQTL explaining too little variance for us to detect in a sample of this size, whilst *cis* effects have relatively larger mono- or oligogenic effects. The effect sizes of our *trans* mQTL also tend to be smaller (Additional file 1: Figure S2), although a minority still have effects exceeding a 10 % difference in proportion methylated. Overall our effect sizes exceeded 2 % difference in proportion methylated for more than 50 % of mQTL and greater than 5 % methylated for more than 25 % of mQTL. Whilst this means that some of our mQTL have a potentially small biological effect, these results will still be of value in Mendelian randomization analysis [25] and analysis of the genetic architecture of DNA methylation.

We observed two interesting patterns when we examined the similarities in genetic effects on methylation at different time points. Firstly, fewer mQTL were discovered at birth and estimates of heritability were lower at birth than in childhood. Secondly, heritability of methylation and the total number of mQTL decrease with increasing age (with the exception of birth). The potential explanations for these observations are that either genetic effects change over time or environmental effects change over time. An increase in overall variation in methylation over time suggests that the latter is more likely, with the environment having a relatively greater influence around birth and then later in life. This is consistent with the postulated period of methylation plasticity in utero and the epigenetic drift observed through the life course. The largest proportion of time point-specific effects were at birth (cord blood), with adolescence and middle age having the lowest proportion. This suggests that a small proportion of mQTL may play a more important role during early development, with their relative importance diminishing later in life.

Regional association patterns for *cis* mQTL varied widely between loci, with some showing distinct blocks of mQTL SNPs and associated CpGs (i.e. SNPs associating with many CpGs within the same LD block but very few outside). This apparent alignment of SNP LD blocks with methylation blocks may simply represent the short-range functional effect of SNPs on CpG methylation, with a number of SNPs in LD affecting a number of nearby CpG sites. Other loci show a less distinct pattern of association, with different CpGs associating with different, but overlapping, sets of SNPs.

We observed some evidence that mQTL are enriched in 5′ and 3′ untranslated regions compared with intronic and intergenic regions, whilst the mQTL-associated CpGs tend to be more frequent upstream of the transcription start site. With respect to CpG islands these mQTL-associated sites are enriched in north and south CpG island shores. Whilst we don’t have other QTL data from the same samples for direct comparison, interestingly Banovich et al. [26] report notable overlaps of mQTL with QTL for other regulatory features such as histone modifications, DNase I hypersensitivity, chromatin access and expression levels. Although we don’t have direct measurements of expression to perform eQTL analyses, we did perform a comparison of our mQTL with the GTEx eQTL data [22]. The approximately 5 % of GTEx eQTL that we report as mQTL is not inconsistent with the observation from a study in fibroblasts that found 12 % of genes with an eQTL have a nearby mQTL [27], although Banovich et al. [26] found that, amongst eQTL within 3 kb of a methylation probe, approximately 25 % were also mQTL.

In an additional analysis we found evidence that some *trans* SNP–CpG associations may be mediated by *cis* methylation. A recent mQTL study with a greater sample size than ours found a limited number of mQTL affecting multiple (up to 19) sites [17]. Although we observed a number of such cases, many *trans* effects do not show evidence of *cis* mediation. However, our limited power to detect such mediation mechanisms and the possibility that measurement error reduces the apparent extent of mediation should be taken into account when interpreting these findings. These observations are consistent with a previous report based on lung tissue [21] and underline the complexity of mQTL effects.

We found no evidence of SNPs affecting methylation at a large number of sites, with the majority of our *trans* mQTL representing just one SNP–CpG association. This contrasts with observations in eQTL analysis which suggest some SNPs impact on the regulation of a large number of transcripts as ‘master regulators’ [28]. The difference between mQTL and eQTL in this context aligns with our observation that there is only limited overlap between published blood eQTL [23] and our blood mQTL (discussed in more detail above). A previously reported study of mQTL and eQTL amongst loci associated with bipolar disorder also found orthogonality between the two types of QTL [29]. Grundberg et al. [10], profiling adipose tissue from 648 twins, revealed that 28 % of CpGs were associated with nearby SNPs and, when overlapping them with adipose eQTL from the same individuals, they found that 6 % of the loci played a role in regulating both gene expression and DNA methylation. In contrast, a systems analysis of ~418 K SNPs with ~23 K CpG sites and ~16 K expression probes using blood from 148 individuals found evidence using *cis* eQTL and mQTL to support a causal model in which genotype affects methylation, which in turn affects expression [30]. However, the same study did not investigate *trans* mQTL. There is, therefore, little evidence at this stage to suggest either that there are mQTL master regulators or that eQTL master regulators act on expression via methylation. Thus, whilst methylation is known to influence gene expression, we find limited overlap between mQTL and published eQTL and no evidence of ‘master regulators’ in mQTL, suggesting a more complex relationship than the simple SNP–methylation–expression model, consistent with the recently reported findings of the Epigenomics Roadmap Consortium [31].

Our results support a stable effect of genetic variation on methylation across the life course, with common SNPs accounting for approximately 20 % of variance in methylation (with the caveat that cord blood is quite different in cellular composition from peripheral blood). One important question is whether mQTL play a functional role in disease. One previous study has indicated potential roles for population-differential mQTL in age of menarche, hepatitis B infection and HIV control [18], whilst another has reported potential contributions of mQTL to autoimmune disease (although they didn’t formally analyse enrichment) [18]. Our analyses suggest that mQTL are relevant to common diseases and traits, accounting for a significant proportion of the heritability of Crohn's disease, hypertension, rheumatoid arthritis and type 1 diabetes. Our observations of a potential role in autoimmune disease seem consistent with those of Lemire et al. [17], although it is crucial to consider the potential that autoimmune genotype may drive measured DNA methylation through cellular heterogeneity. The enrichment of mQTL in autoimmune diseases is unlikely to be driven by large effects and long-range LD in the major histocompatibility complex (MHC) region because reanalysis excluding chromosome 6 returned the same conclusion. Whilst this observation does not prove causality, it suggests two important phenomena. First, it provides a plausible role of methylation in mediating some of the effects of common genetic variation on disease and is consistent with one of the first mQTL analyses to use the Illumina Infinium HM450 array, which demonstrated enrichment of mQTL among disease-associated loci [21]. Second, it suggests that if methylation influences complex traits, then this is likely through variation at many genomic loci, with each locus having a small effect.

### Limitations

Methylation data were only available in peripheral blood from ALSPAC participants, so we were unable to compare results between tissues (interestingly, mQTL have been reported to be enriched for CpG sites that are variable between tissues [32]). In addition, cord blood is not directly equivalent to peripheral blood. Cellular heterogeneity can account for differences in methylation between samples, affecting different CpG sites to different extents; we had no directly measured cell count data available but applied an established method [33] to adjust for the potential confounding influence of these effects. However, this method was optimised on adult samples (not children). It may also not be an appropriate approach for use in cord blood, which may explain some of the differences we observe between birth and other time points.

DNA was extracted from different blood sample types, which could potentially affect methylation measurements, although in seeking to test this possibility we illustrate in Additional file 1: Figure S17 that sample type does not appear to stratify the top principal components of DNA methylation.

The adults in our sample are all female, so one may wish to consider the generalizability of the reported findings to adult males when interpreting data.

Some probes on the Illumina Infinium HM450 array are known to be affected by a range of factors, including underlying SNPs [34]; we filtered out the most severely affected but recommend that this is taken into account when interpreting and subsequently utilising the data we have generated.

Whilst the number of mQTL we report in Table 1 differs from those in other publications (varying from 49 [35] to 52,708 [17] or more *cis* mQTL), it is important to note that these numbers rely on arbitrary thresholds, are susceptible to power and heavily influenced by definitions of *cis* and *trans*.

We note that even though there was generally good agreement in overall results between our two null models (null SNPs sampled from genic regions and null SNPs sampled from the same distribution of genic annotations as the true mQTLs), the enrichment *p* values were quite sensitive to the null model used. We recommend caution in performing and interpreting such enrichment analyses.

## Conclusions

We demonstrate that genetic effects on methylation levels are highly stable over time and that methylation variation increases over time, most likely due to increased environmental or stochastic influences. We also provide evidence that methylation may play a mediating role in the influence of genetic variation on complex traits. We are releasing the results from our mQTL analysis in the form of an open database (http://www.mqtldb.org/) to encourage consistent publication and integration of results from other studies.

## Methods

### Study sample

Samples were drawn from the Avon Longitudinal Study of Parents and Children [12, 13]. Blood from 1018 mother–child pairs (children at three time points and their mothers at two time points) were selected for analysis as part of the Accessible Resource for Integrative Epigenomic Studies (ARIES, http://www.ariesepigenomics.org.uk/) (Additional file 1: Table S1) [14]. Numbers of samples surviving the imposed quality control (QC) thresholds at each time point are shown in Additional file 1: Table S2. Sample selection was on the basis of sample availability at all of the chosen time points in mother–child pairs. Cord blood and peripheral blood samples (whole blood, buffy coats, white blood cells or blood spots) were collected according to standard procedures.

### Methylation assays

Following DNA extraction, samples were bisulphite converted using the Zymo EZ DNA Methylation™ kit (Zymo, Irvine, CA, USA). Following conversion, genome-wide methylation was measured using the Illumina Infinium HumanMethylation450 (HM450) BeadChip. The arrays were scanned using an Illumina iScan, with initial quality review using GenomeStudio. During the data generation process a wide range of batch variables were recorded in a purpose-built laboratory information management system (LIMS). The LIMS also reported QC metrics from the standard control probes on the HM450 BeadChip for each sample. Samples failing QC were excluded from further analysis and the assay repeated. Data points with a low signal:noise ratio (detection *p* > 0.01) or with methylated or unmethylated read counts of 0 were also excluded from analysis. As an additional QC step genotype probes on the Ilumina Infinium HM450 array were compared between samples from the same individual at different time points and against SNP-chip data (HM450 probes clustered using k-means) to identify and remove any sample mismatches. Methylation data were normalised in R with the wateRmelon package [36] using the Touleimat and Tost [37] algorithm to reduce the non-biological differences between probes. Data were then rank-normalised to remove outliers and regressed on all covariates plus bisulphite-converted DNA (BCD) plate batch to remove potential batch effects (with missing values set to probe mean).

### Genotyping assays

The ARIES participants were previously genotyped as part of the larger ALSPAC study, with QC, cleaning and imputation performed at the cohort level before extraction of the subset comprising ARIES. ARIES participants are of European white ancestry and homogeneous compared with HapMap reference populations (Additional file 1: Figure S18).

Children were genotyped using the Illumina HumanHap550 quad genome-wide SNP genotyping platform (Illumina Inc., San Diego, CA, USA) by the Wellcome Trust Sanger Institute (WTSI; Cambridge, UK) and the Laboratory Corporation of America (LCA, Burlington, NC, USA). Individuals were excluded on the basis of incorrect gender assignment, abnormal heterozygosity (<0.320 or >0.345 for WTSI data; <0.310 or >0.330 for LCA data), high missingness (>3 %), cryptic relatedness (>10 % identity by descent) and non-European ancestry (detected by multidimensional scaling analysis). Following QC, the final directly genotyped dataset contained 500,527 SNP loci.

Mothers were genotyped using the Illumina human660W-quad genome-wide SNP genotyping platform (Illumina Inc., San Diego, CA, USA) at the Centre National de Génotypage (CNG; Paris, France). Individuals were excluded based on non-European ancestry, missingness, relatedness, gender mismatches and heterozygosity. PLINK (v1.07) [38] was used to carry out quality control measures on an initial set of 10,015 subjects (including non-ARIES ALSPAC participants) and 557,124 directly genotyped SNPs. Following QC, the final directly genotyped dataset contained 526,688 SNP loci.

Imputation was performed to increase the SNP density for all genotyped mothers and children combined. Genotypes were phased together using ShapeIt (version 2, revision 727) and then imputed against the 1000 Genomes reference panel (phase 1, version 3, phased using ShapeIt version 2, December 2013, using all populations) using Impute (v2.2.2). Genotypes were filtered to have Hardy–Weinberg equilibrium *p* > 5 × 10^−7^, MAF >1 % and imputation info score >0.8. Best guess genotypes were used for subsequent analysis. The final imputed dataset used for the analyses presented here contained 8,074,398 loci.

### Genotype/methylation association tests

Summary details of samples, SNPs, CpGs and covariates included in association tests are presented in Additional file 1: Table S2. Each SNP in the imputed datasets was analysed against all CpG sites in the Illumina Infinium HM450 array with the exception of those failing QC and those reported to map to more than one location (N = 19,834) or to contain a genetic variant at the CpG site (N = 74,182) [34]. We performed a post hoc test for analysis of non-specific probes which determined that there was no enrichment of cross-hybridising probes in our results. We opted for post hoc annotation of potential probe effects within our results database for the remaining potentially problematic CpG sites (N = 229,983) [34]. The final number of probes analysed was 395,625. Preliminary association analysis of SNPs with CpG sites was performed using an additive model (rank-normalised CpG methylation on SNP allele count) using Matrix eQTL [39] in order to perform the computationally demanding task of estimating 16 trillion associations. Analyses were batched by SNP chromosome and sample time point for parallel analysis on the University of Bristol High Performance Computing (HPC) cluster. SNP effects from this analysis that were p < 1 × 10^−7^ were then taken forward for re-analysis in PLINK1.07 to perform exact linear regression including covariates. Covariates included in all analyses were age (excluding birth), sex (children only), the top ten ancestry principal components, bisulphite conversion batch and estimated white blood cell counts (using an algorithm based on differential methylation between cell types [33]). Methylation at each CpG site was regressed on these covariates and residuals taken forward for regression on SNP genotype. To report the number of mQTL, we used a conservative threshold of 1 × 10^−14^. All associations below 1 × 10^−7^ were stored and are available in our online mQTL database (http://www.mqtldb.org/). Analysis on rank-normalised data results in effect sizes that are not directly interpretable on the original scale. Additional file 1: Figure S2 illustrates the effect size distributions observed in our data.

Without access to an appropriate replication sample, we performed all subsequent enrichment and downstream analysis using only *cis* mQTL with *p* < 1 × 10^−14^ (Additional file 1: Table S3) to reduce the possibility of including false positives (unless otherwise stated). We consider results below *p* < 1 × 10^−14^ in a single time point to provide informative evidence of association on the basis that this corresponds to a 0.2 % false positive rate after a Bonferonni correction for the number of tests based on directly genotyped SNPs and directly assayed CpG sites. We define replication in additional time points to be associations at *p* < 1 × 10^−7^ because typically we are testing in the order of 30,000 associations for replication and on the basis that these are supported by their combination with the evidence at other time points through the life course.

### Code availability

Code to run comparable mQTL analyses with ARIES data is available under the GNU GPL (v3) license at https://github.com/MRCIEU/ariesmqtl.

### Conditional mQTL analysis

Linkage disequilibrium is expected to cause many mQTL to be represented by multiple tag SNPs. We used the conditional analysis implementation in GCTA [19, 40] to determine the most representative independent loci associated with each CpG site. We used the summary statistics from the results of our PLINK analysis (i.e. any SNP–CpG pair with a *p* ≤ 1 × 10^−14^ at any time point) and entered them into a stepwise model using individual level genotype data to determine whether they independently account for association with the CpG site. Independent signals with *p* < 1 × 10^−14^ are considered significant.

### Patterns of methylation across genomic features and time

The distribution of DNA methylation across CpG sites in different genic and CpG island regions was analysed using annotations from the Illumina Infinium HM450 manifest file (v1.2). Each probe was assigned to an annotation category (e.g. 5′ UTR, 3′ UTR, gene body, CpG island, etc.), the median methylation beta (proportion methylation) for that probe was calculated and then the distribution of these medians across mQTL-associated CpG sites in a category was plotted in a histogram.

To characterise the temporal pattern of methylation, the correlation of methylation between each pair of time points was calculated for each probe. The distribution of correlation coefficients (r^2^) was plotted in a histogram for each pair of time points. A null distribution of correlation for each pair was derived by randomising the sample order for one of the pair of time points, and similarly plotted as a histogram of correlation coefficients.

### Patterns of mQTL across genomic features and time

The distribution of mQTL-associated CpG sites was analysed using annotations from the Illumina Infinium HM450 manifest file (v1.2). The proportion of CpG probes falling into each annotation category was plotted in a bar chart to evaluate the distribution across different genomic features. A similar approach was used for mQTL SNPs, which were annotated with features using the Variant Effect Predictor [41] (all associated SNPs were included).

### SNP heritability estimation of methylation probes

In order to estimate the proportion of the variation in CpG probes that is due to genetic variation, we used SNP heritability analysis for each probe (after filtering) at each time point. This was performed using genomic restricted maximum likelihood (GREML) as implemented in GCTA (v1.24). The same covariates were fitted here as they were for the mQTL mapping analysis. Only unrelated individuals were used in these analyses (genetic relatedness <0.05) in order to obtain a population-based ‘SNP’ heritability estimate, which is typically an underestimate of true heritability in complex traits because the magnitude of the estimate is limited to the proportion of all genomic variation that is captured by the SNPs on the genotyping array. In this case we used HapMap3 [20] SNPs with MAF >0.01 and imputation quality score >0.8 (1,171,463 SNPs after filtering). The SNP heritability was estimated for each probe using two variance components, the first generated using only SNPs within ±1 Mb of the CpG site (the *cis* component) and the second generated using all remaining SNPs (the *trans* component), such that var(y) = var(g_cis) + var(g_trans) + var(e), where the genetic variance due to all SNPs var(g) = var(gi_cis) + var(g_trans), and var(g)/var(p) is the total SNP heritability for a probe at a particular time point. The same analysis was repeated for each probe at each time point.

We then make a distinction between ‘explained’ and ‘unexplained’ genetic variation (Fig. 2b). Some proportion of the total estimated SNP heritability has been ‘explained’ by the detected mQTLs, estimated by summing the R^2^ values for each conditionally significant association at *p* < 1 × 10^−14^ for a particular probe at a particular time point. Thus, we term the remaining genetic variation ‘unexplained’. The ‘explained’ genetic variation was estimated for both the *cis* and *trans* genetic components.

### Proportion of trait variance explained by mQTL

In order to ascertain if mQTL contribute to variance in disease liability, a mixed model analysis was performed to calculate the proportion of the trait variance that could be explained by mQTL and non-mQTL in publicly available data from the first WTCCC publication [24]. The variance partitioning model:

$$ {y}_i=\mu +{g}_{i,1}+{g}_{i,2}+{e}_i $$

was fitted using REML as implemented in GCTA (v1.24) software [19]. Here, *y*_*i*_ is the phenotype of individual *i*, *g*_*i,*1_ is their genetic value due to mQTL, *g*_*i,*2_ is their genetic value due to all HapMap3 [20] SNPs that were not identified as mQTL and *e*_*i*_ is their residual value. To avoid results being influenced by MHC, both *g*_*i,*1_ and *g*_*i,*2_ were estimated excluding all SNPs on chromosome 6. This analysis was performed for seven traits (bipolar disorder, Crohn’s disease, type 1 diabetes, type 2 diabetes, coronary heart disease, hypertension, rheumatoid arthritis) using the WTCCC data [24]. The WTCCC data underwent the same quality control procedure described in [42]. Specifically, the data were split into seven case-control datasets where the controls were common across all traits; each dataset was filtered for MAF >0.01, genotype missingness <0.002, Hardy–Weinberg equilibrium *p* > 0.01 and any SNPs with differential missingness between cases and controls (*p* < 0.05) were removed. This left approximately 230,000 SNPs for each dataset. Pairwise genomic similarity was then estimated using scaled SNP similarity and any individual pairs with genomic relatedness >0.025 were removed. Principal components were estimated from the SNP data after removing regions of long-range LD and pruning to low LD and five rounds of outlier removal were used whereby if any individual had a value of mean ± 5 standard deviations in any of the first 20 components they were removed. Imputation was performed to 1000 Genomes reference panel (phase 1, version 3, release December 2013) in two stages: first, haplotypes of the WTCCC samples were estimated jointly using ShapeIt2 and then imputation was performed using Impute2. Then, *g*_*i,*2_ was estimated using only the imputed SNPs that were present in the HapMap3 reference. To test if the mQTLs detected in ARIES made disproportionately large contributions to SNP heritability in the WTCCC datasets, we used only *cis* mQTL with *p* < 1 × 10^−14^ at any time point that were then LD pruned, leaving 29,805 SNPs in total. Generating an appropriate null distribution against which to compare the point estimate of the mQTL variance component in this analysis is important because the mQTL variance component may have been contaminated by variance due to genic SNPs rather than directly due to mQTL alone. To test for this possibility, we generated two different ‘null’ experiments. First, we sampled 29,805 SNPs that were within ±500 kb of a gene (‘genic SNPs’), matching the SNPs to have the same LD and allele frequency distributions as the mQTLs and performing the entire analysis again, but instead of using mQTLs, *g*_*i,*1_ was constructed using these sampled SNPs. This was performed 100 times to get a distribution of estimates under the null hypothesis that proximity to genic regions is sufficient to explain the influence of mQTLs on the trait. Second, the same was done except instead of sampling from genic regions specifically, the null SNP set was generated to have the same number of SNPs in 5′ UTR, 3′ UTR, intronic, intergenic, upstream, downstream and unannotated regions of the genome (‘mQTL annotation-matched SNPs’). All SNP heritability estimates were transformed from the observed to the liability scale using the trait prevalence estimates used in [43]. Sex and the first 20 genetic principal components were included as covariates in all analyses.

### Mediation of *trans* mQTL effects by *cis* methylation

Potential mediation of *trans* mQTL effects by *cis* methylation has been previously reported [21]. This type of analysis is particularly susceptible to measurement error in the *cis* (mediating) CpG methylation variable so requires cautious interpretation. For all *trans* associations we took the estimate of association from a linear regression of B ~ G, and then performed a second linear regression B ~ G + Kx, where B is methylation at the *trans* CpG site, G is the genotype (or allele score if more than one independent *cis* association) and K is methylation at the *cis* CpG site. Reduction in regression coefficient for genotype when *cis* CpG methylation was added to the model was evaluated as an estimate of potential mediation (given the limitations of measurement error). We estimated the likely impact of measurement error by performing a series of analyses in which we added increasing levels of simulated measurement error (multiplication by random values sampled from a normal distribution with mean 1 and a given standard deviation).

### Enrichment analysis for mQTL in GWAS results

To test if the mQTL were enriched for low *p* values in previously published GWAS results, the following procedure was performed. Only independent mQTL with *cis* effects of *p* < 1 × 10^−14^ obtained from the conditional analysis and then filtered to remove SNPs with LD r^2^ > 0.1 were used to test for enrichment. We collected data from a set of 33 complex traits from online sources. All available mQTL were extracted from each trait and Fisher's method was used to estimate a combined *p* value for each trait:

$$ {\chi}_{2k}^2 \sim - 2{\displaystyle \sum_{i=1}^k \ln\ {p}_i} $$

In order to compare these *p* values to a realistic null distribution, the same procedure was performed but using 10,000 random draws of the same number of SNPs from (a) genic regions or (b) mQTL annotation-matched SNPs. In both cases SNPs were also matched to the mQTLs on allele frequency and number of proxies to adjust for LD structure. In order to match on LD, each mQTL was given an LD score by calculating the number of SNPs that were in LD r^2^ > 0.8 and the same was done for each of the candidate genic SNPs (approximately 200,000 genic SNPs). A null distribution for each GWAS trait was generated by performing this procedure 1000 times, each time making a new random draw of genic SNPs matched for allele frequency and LD. Empirical *p* values were generated by simply finding the rank of the meQTL *p* value among the 1000 null *p* values.

### Gene ontology enrichment

mQTL SNPs were mapped to the nearest gene (by genomic coordinate) using the Variant Effect Predictor and lists of unique gene names were analysed for enrichment of gene ontology (GO) terms relative to a human reference using the gene ontology function (‘go’) in the Orange bioinformatics add-on (‘Orange.bio’ , http://orange.biolab.si/download/). CpG sites associated with mQTL were similarly (but separately) tested for GO term enrichment using the Illumina gene-name annotations for each CpG site. Analyses were repeated for time point-specific loci and those common to all time points. Potentially enriched GO terms (biological function) were plotted using REVIGO [44], where strength of evidence for enrichment (−log_10_(*p* value)) is proportional to size.

### Comparison of blood mQTL with blood eQTL

Previously reported blood eQTL [22] were downloaded from http://www.gtexportal.org/ and compared with mQTL discovered at each time point to determine overlap. *Cis* QTL were considered to be shared if the same SNP was reported to be associated at *p* < 1 × 10^−14^ in mQTL data and *p* < 2.5 × 10^−7^ in eQTL data (the reported GTEx threshold). For GO analyses we compared our mQTL data with blood eQTL from Westra et al. [23] as these data enabled us to evaluate enrichment of common QTLs for both *cis* and *trans*; however, a lack of data prevented us from comparing the numeric overlap between the Westra et al. eQTL and our mQTL.

### Ethics approval and consent to participate

Written informed consent has been obtained from all ALSPAC participants. Ethical approval for the study was obtained from the ALSPAC Ethics and Law Committee (IRB00003312) and Local Research Ethics Committees in accordance with the guidelines of The Declaration of Helsinki.

### Availability of data and materials

A mQTL database containing all results from this study is available online at http://www.mqtldb.org, which includes both search functionality and full download of all results at *p* < 1 × 10^−7^ using Matrix eQTL and GCTA. Data are also available for download at doi:10.5523/bris.r9bxayo5mmk510dczq6golkmb.

All data used in this study were provided to the authors by the ALSPAC cohort study via their standard Access Policy. Data are available to other bona fide researchers on the same basis; for details please see http://www.bristol.ac.uk/alspac/researchers/data-access/.

## Additional file

Additional file 1: Supplemental data including Figures S1–S18 and Tables S1–S3. (DOCX 12388 kb)
