# Supplementary material for: Systematic identification of genetic influences on methylation across the human life course
Source: Genome Biol. 2016 Mar 31;17:61. doi: 10.1186/s13059-016-0926-z (PMC4818469; doi:10.1186/s13059-016-0926-z)
Supplement: Additional file 1: — Supplemental data including Figures S1–S18 and Tables S1–S3. (DOCX 12388 kb) [file 13059_2016_926_MOESM1_ESM.docx]

# Supplemental Data

Gaunt *et al: “*Systematic identification of genetic influences on methylation across the human life course”

**Figure S1:** Top: The distribution of primary (f1) and additional (f2-5) mQTL identified after performing conditional analysis. Bottom: The distribution of independent trans mQTL identified using clumping.


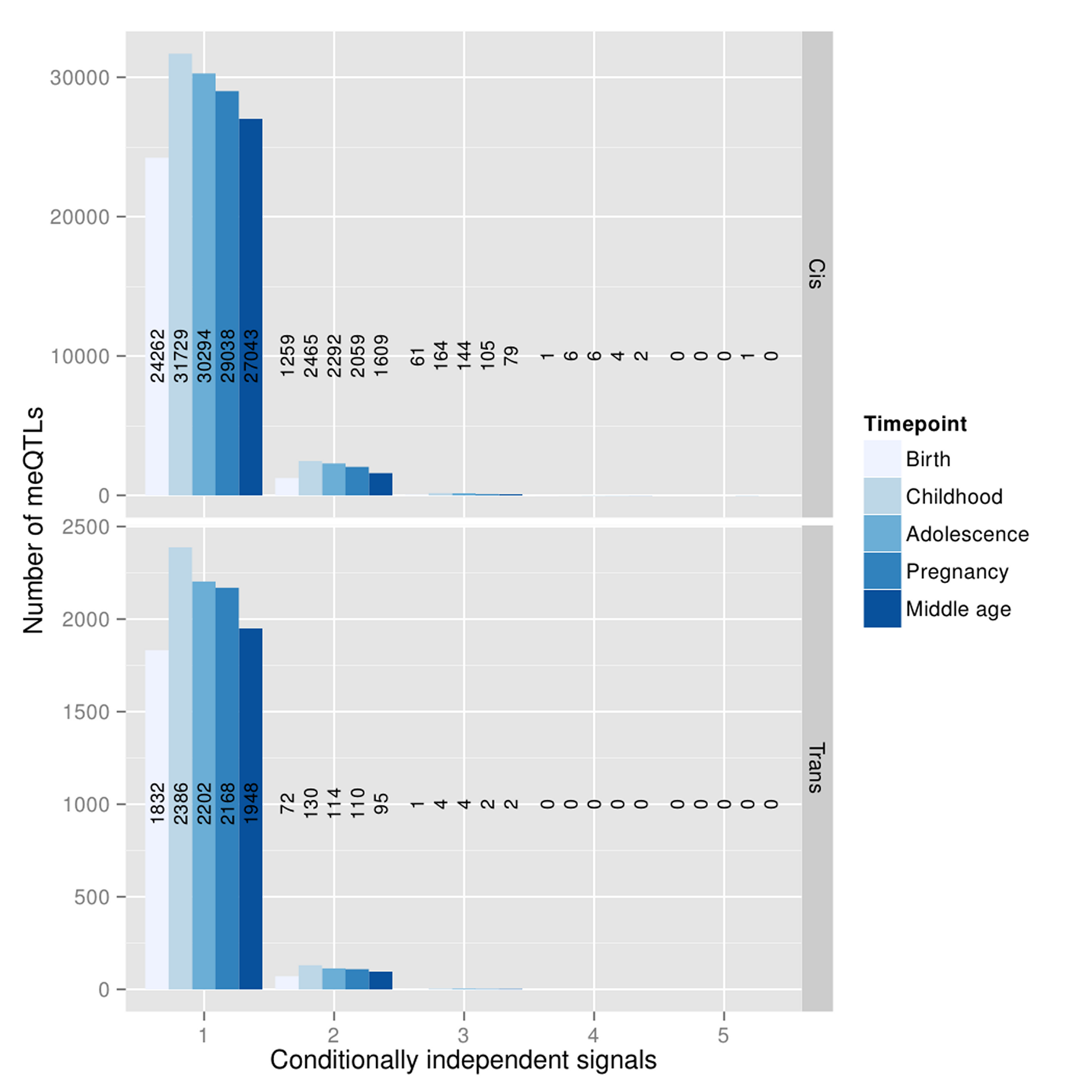


**Figure S2:** Effect size distributions. **A** illustrates effect size distributions for all (black), cis (red) and trans (blue) mQTL calculated by dividing the absolute difference in median beta between opposite homozygotes by 2 (to get an approximation of per allele difference in proportion methylation). **B** provides a categorical presentation of the same data.


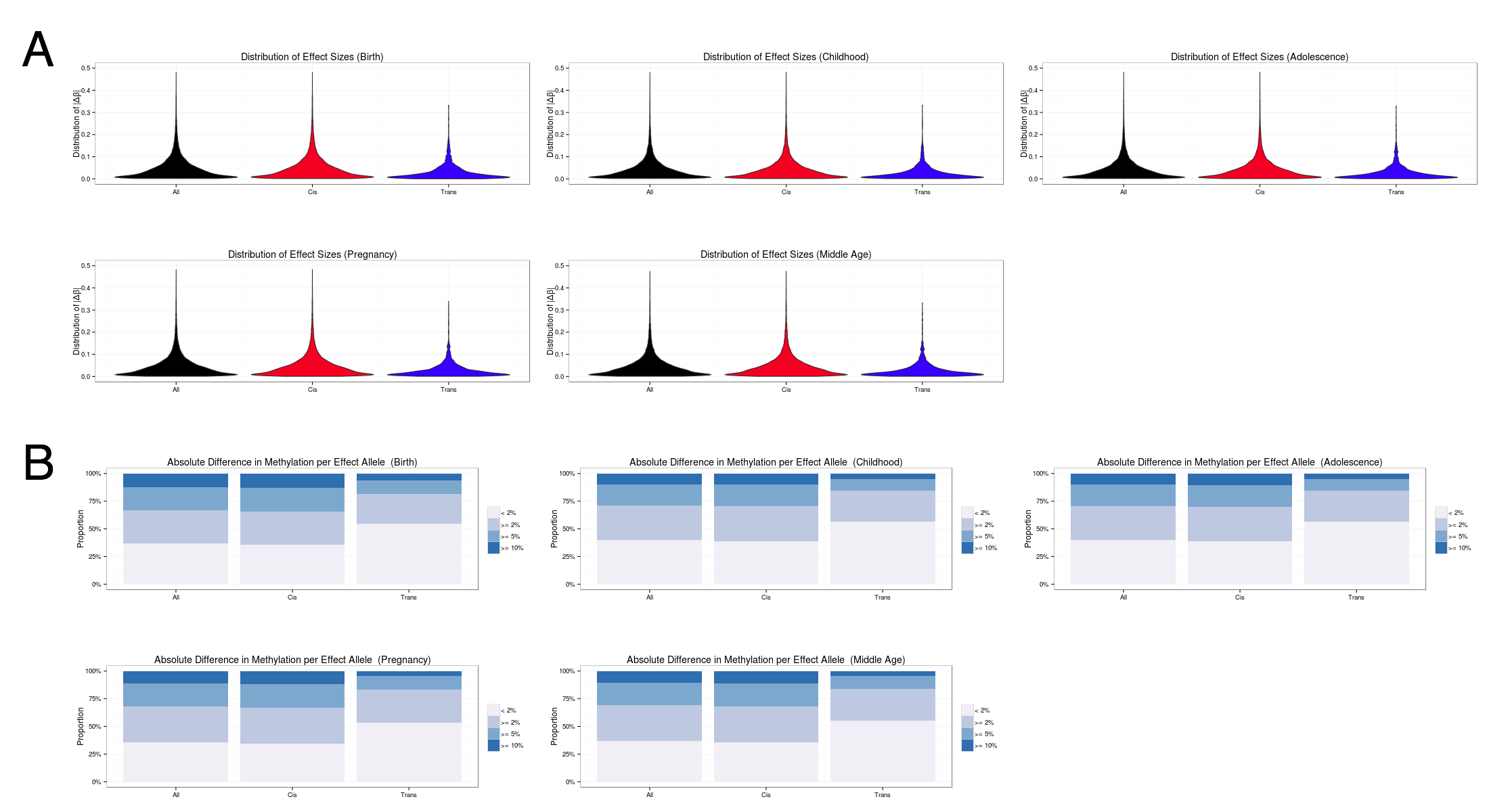


**Figure S3:** Replication of (I) all, (II) cis and (III) trans SNP-CpG associations across time points. Time points are B=birth, C=childhood, A-adolescence, P=pregnancy and M=middle age. The outer lobes show time point-specific associations, while the central portion illustrates associations common to all time points.


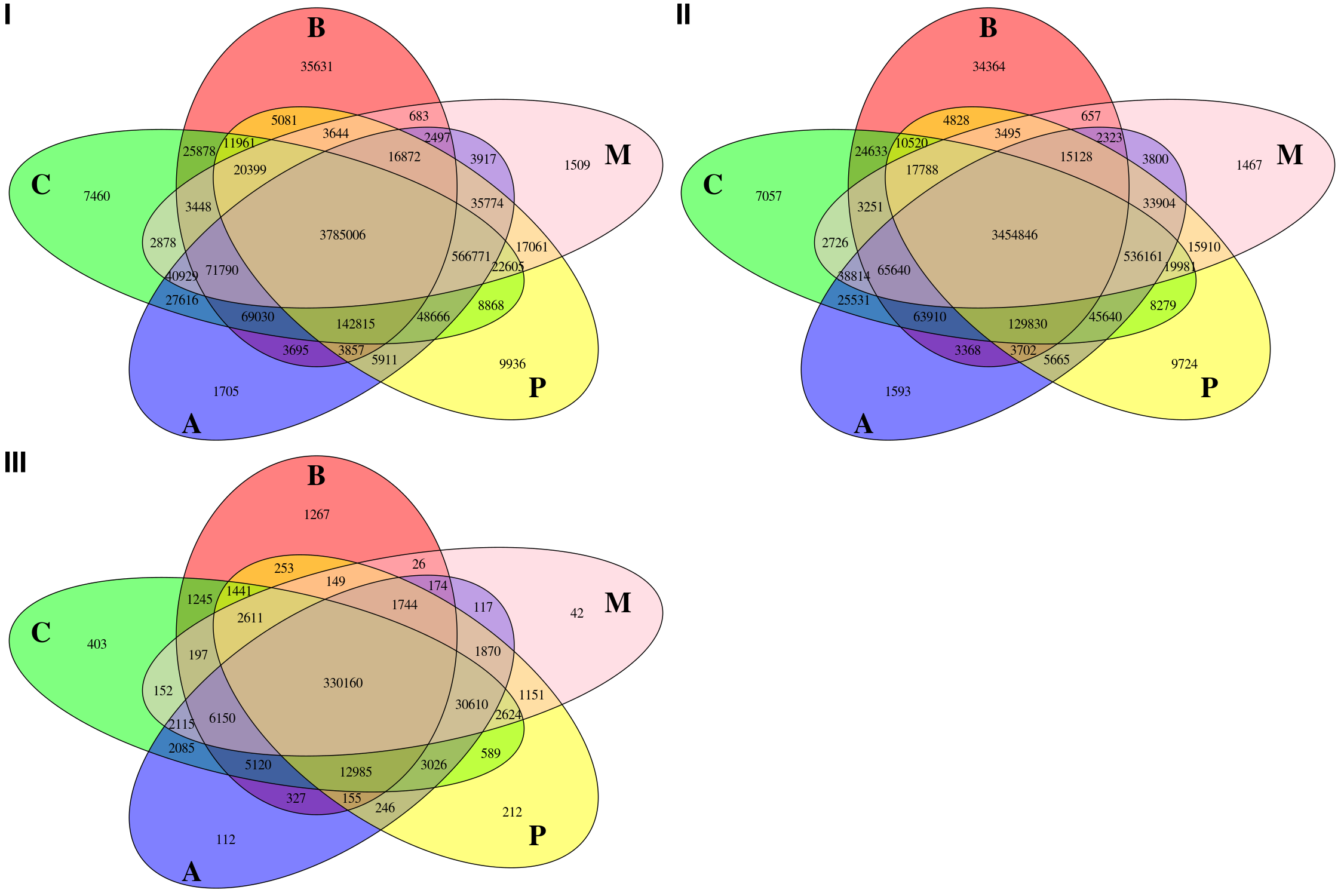


**Figure S4:** Distribution of correlation between time points at each measured CpG site (black) compared to a null model (red) in which sample order was randomised for one of the pair of time points. x-axis = correlation coefficient (r^2^), y-axis = density. Top/right plots show methylation beta values (proportion methylated), bottom/left show rank normalized methylation data.


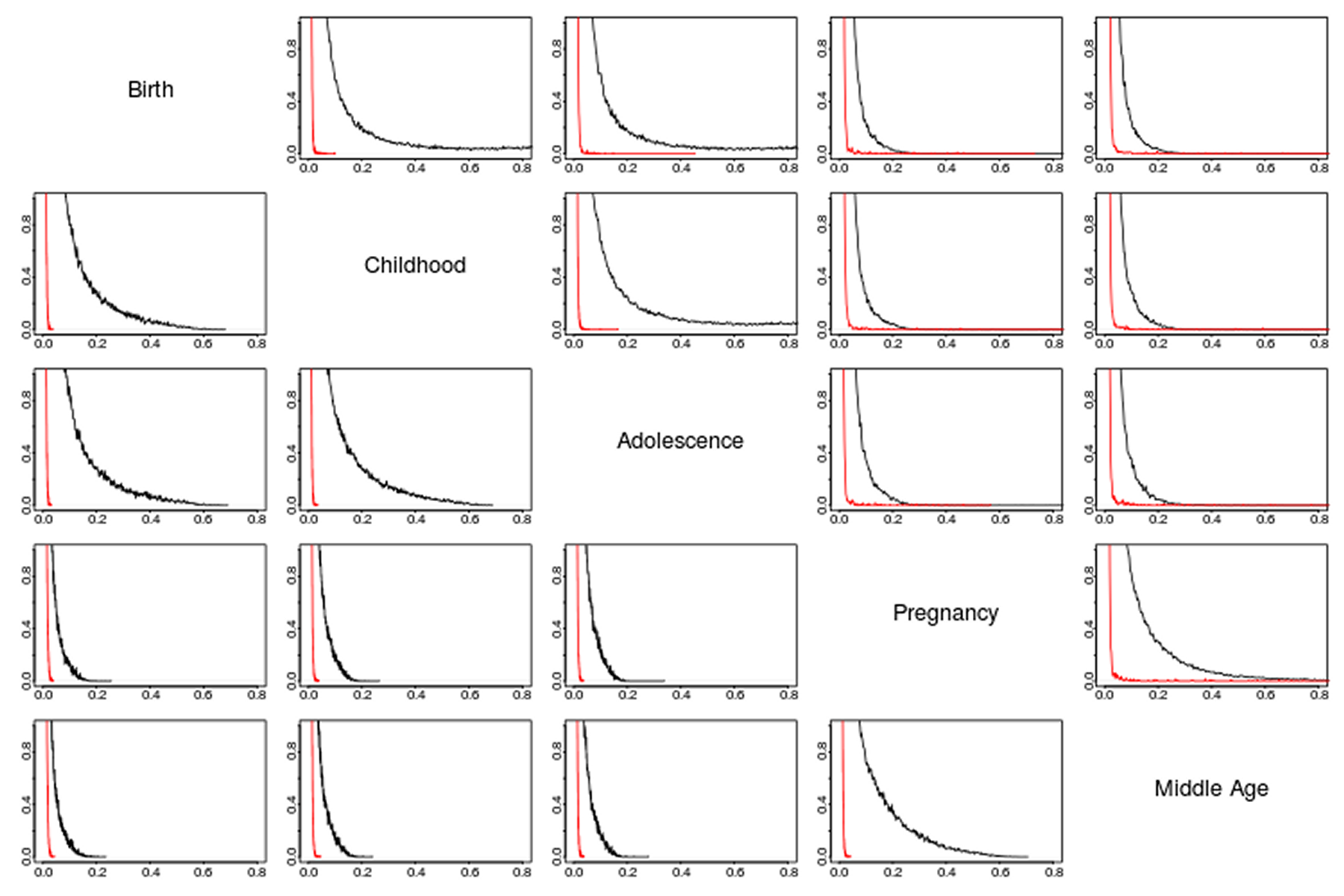


**Figure S5:** Distribution of correlations of CpG methylation levels between childhood and adolescence.

**Figure S6:** Circos plots illustrating trans meQTL across the genome at all five time points. B Birth; C Childhood; A Adolescence; P Pregnancy; M Middle age. From the outside inwards: chromosomes, -log10 (p-value) for association (red points), density of associated SNPs (blue bars), density of associated CpGs (green bars), associations between SNP and CpG (lines).


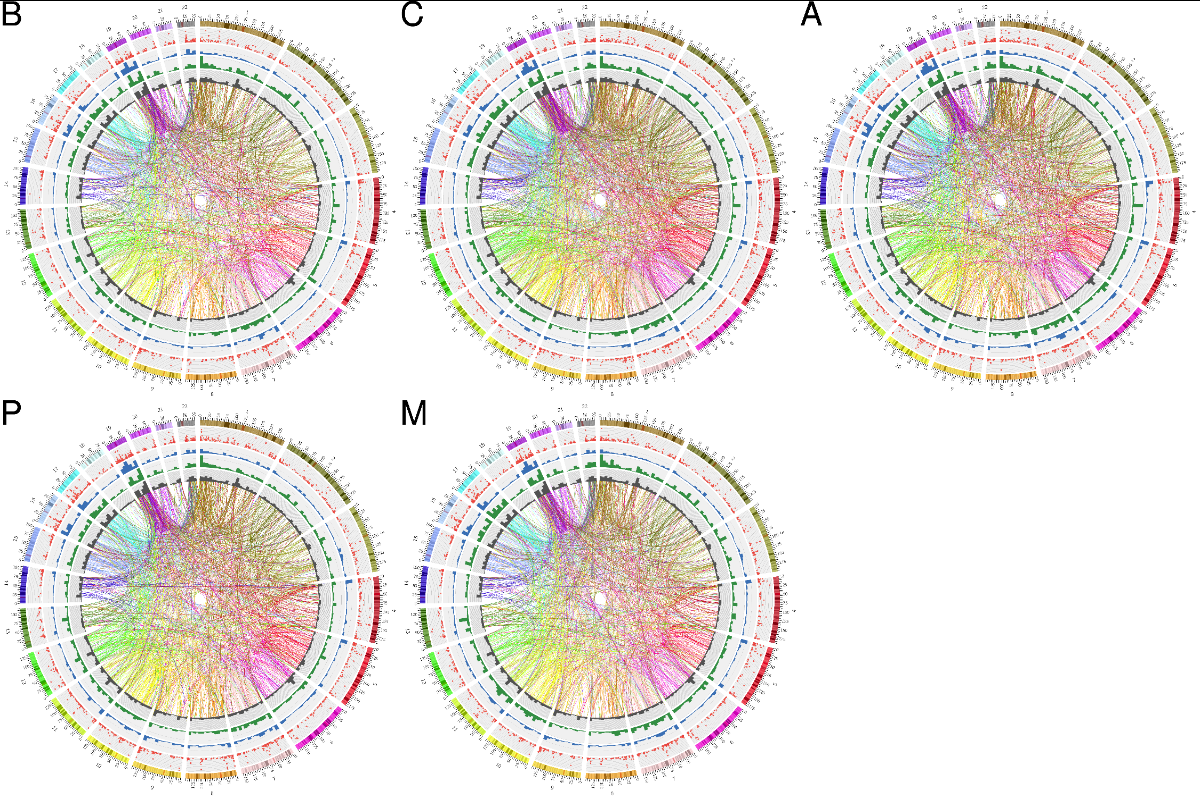


**Figure S7:** Analysis of mediation of trans mQTL effects by cis CpG sites in middle age. (A) Scatter plot showing trans mQTL associations: x-axis = trans mQTL regression coefficient (trans CpG methylation regressed on SNP genotype), y-axis = trans mQTL regression coefficient adjusted for cis methylation (trans CpG methylation regressed on SNP genotype and cis CpG methylation). Error bars show 95% CIs, with gray colour indicating intersection of 95% CIs with x=y line. (B) Models of mediation: in which SNP (S) acts on cis and trans CpG sites either independently, or via mediation.


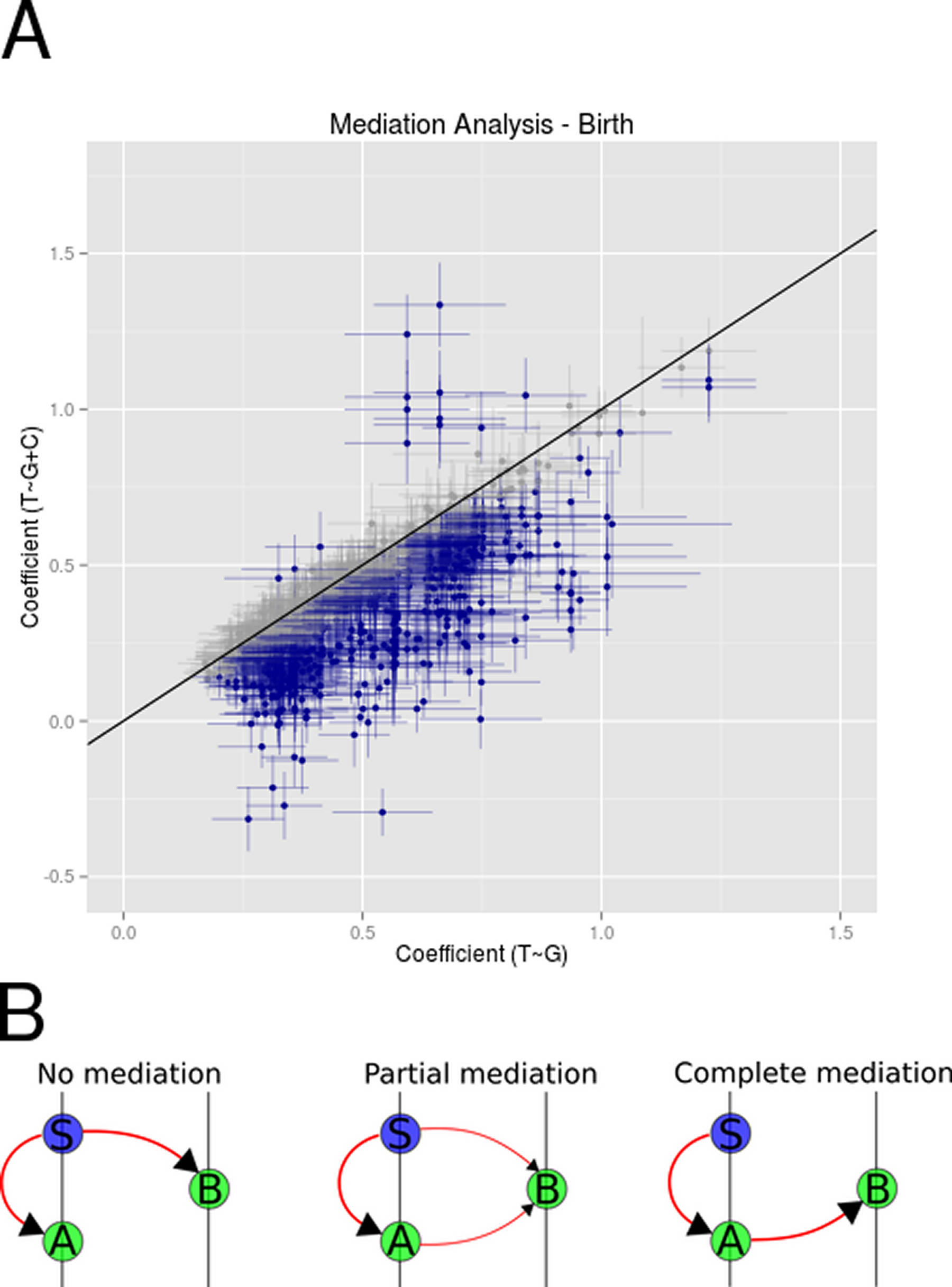


**Figure S8:** Influence of measurement error on mediation analysis. The ratio of regression coefficient from (transCpG~SNP+cisCpG) to the regression coefficient (transCpG~SNP) shows the proportion of trans-meQTL effect that is independent of cis-meQTL. Each line represents differing degrees of artificial noise (simulated measurement error) added to cisCpG methylation. Under the NULL model cisCpG methylation is random.


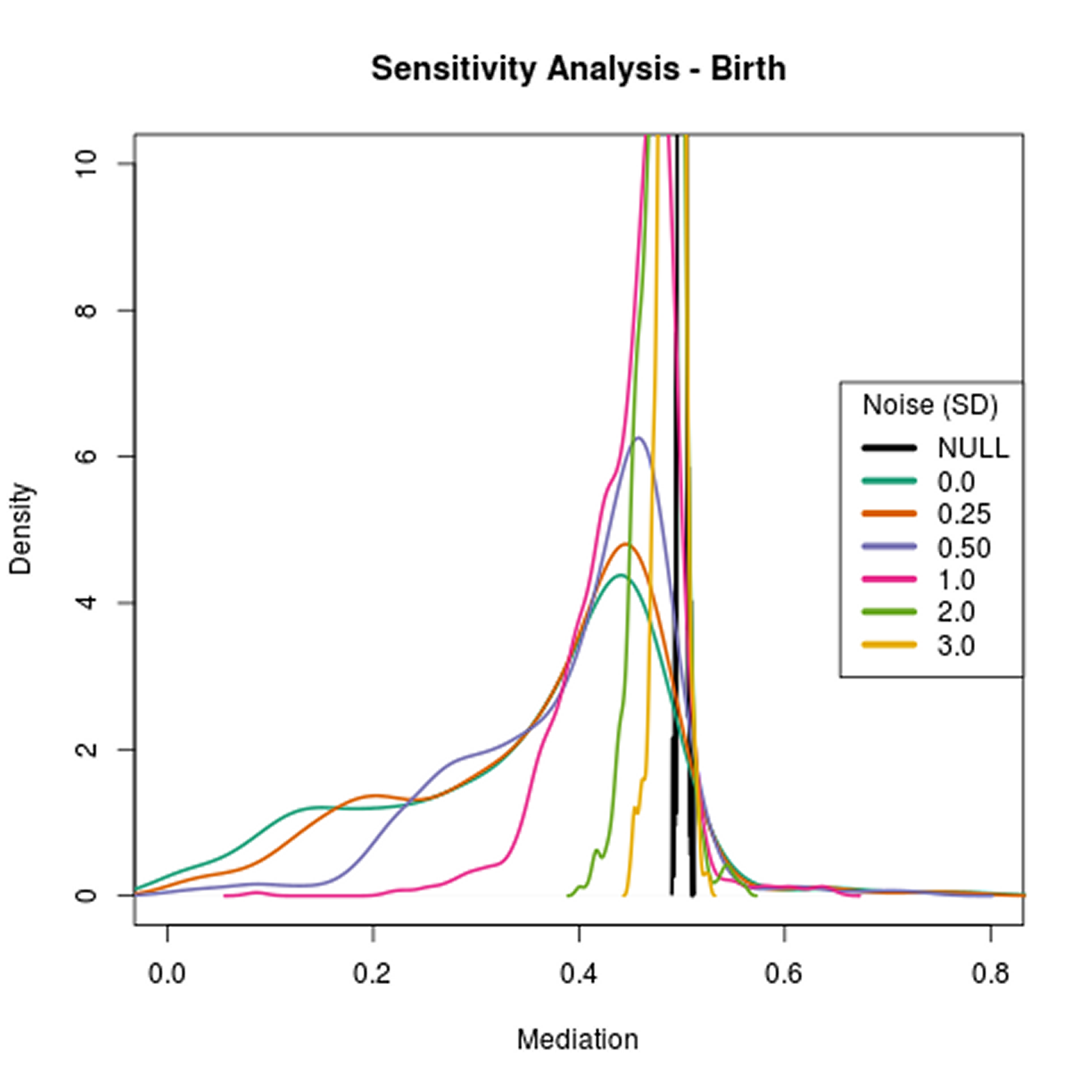


**Figure S9:** Distributions of proportion of DNA molecules methylated at each CpG site according to genic location of CpG site. X axis is proportion methylated, y axis is the proportion of CpG sites in each bin. Plots are shown for both cis (black) and trans (red) associated CpGs at Birth (A), Childhood (B), Adolescence (C), Pregnancy (D) and Middle age (E).


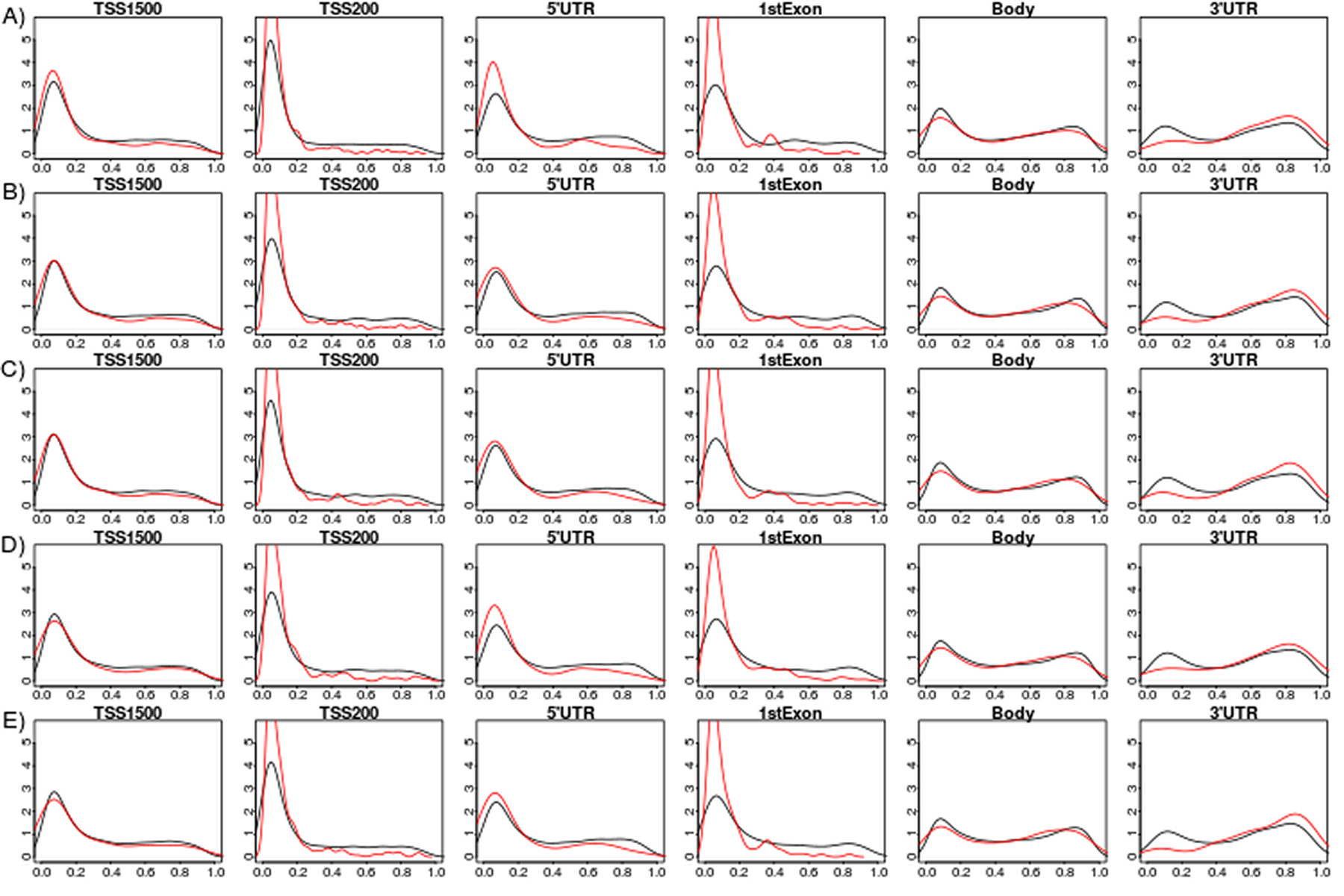


**Figure S10:** Distributions of proportion of DNA molecules methylated at each CpG site according to location of CpG site relative to CpG islands. X axis is proportion methylated, y axis is the proportion of CpG sites in each bin. Plots are shown for both cis (black) and trans (red) associated CpGs at Birth (A), Childhood (B), Adolescence (C), Pregnancy (D) and Middle age (E).


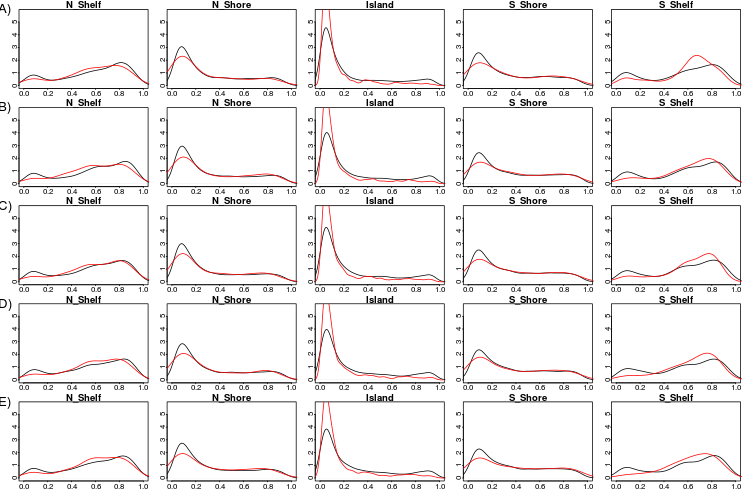


**Figure S11:** 2-dimensional regional association plots for the top 25 cis associated regions (across all time points). These plots show SNP position (x axis) vs CpG position (y axis). Red blocks are the regions spanned by genes (named at center of block). Associations are represented by black dots, with size proportional to -log_10_(p-value). Plots are centered at genomic coordinate of the associated CpG site, with this location indicated by red lines. Order of plots (left to right) is birth, childhood, adolescence, pregnancy and middle age. Order of plots (top to bottom) is in order of ascending p-value across all time points.

1: cg11281224


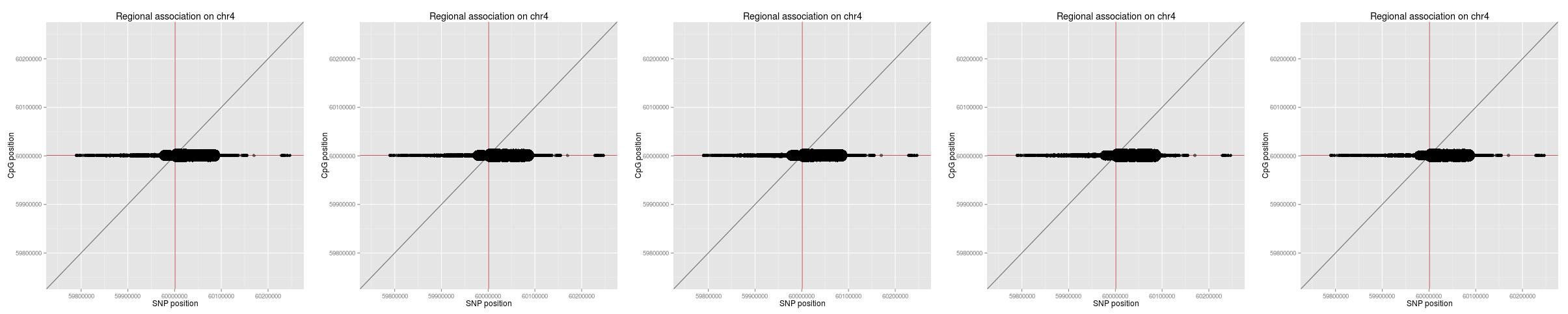


2: cg21069494


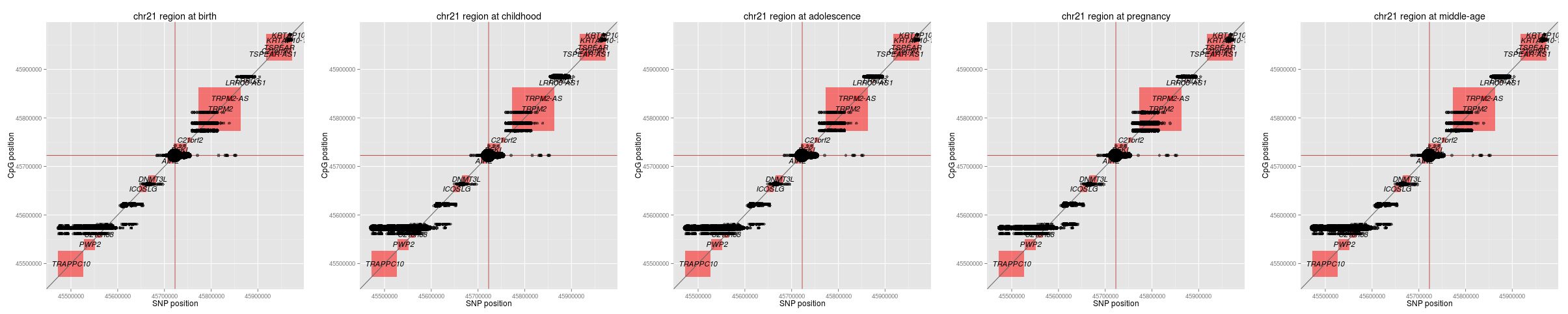


3: cg23649088


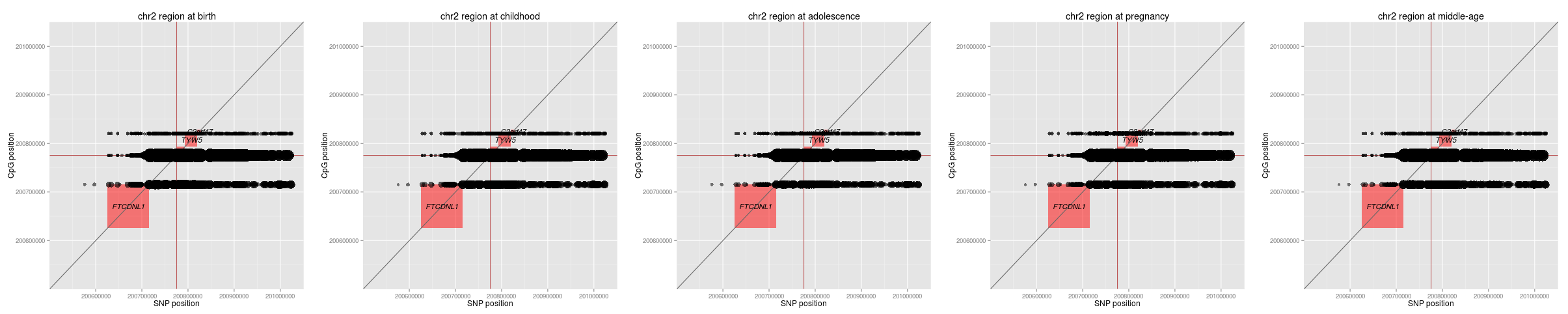


4: cg06713675


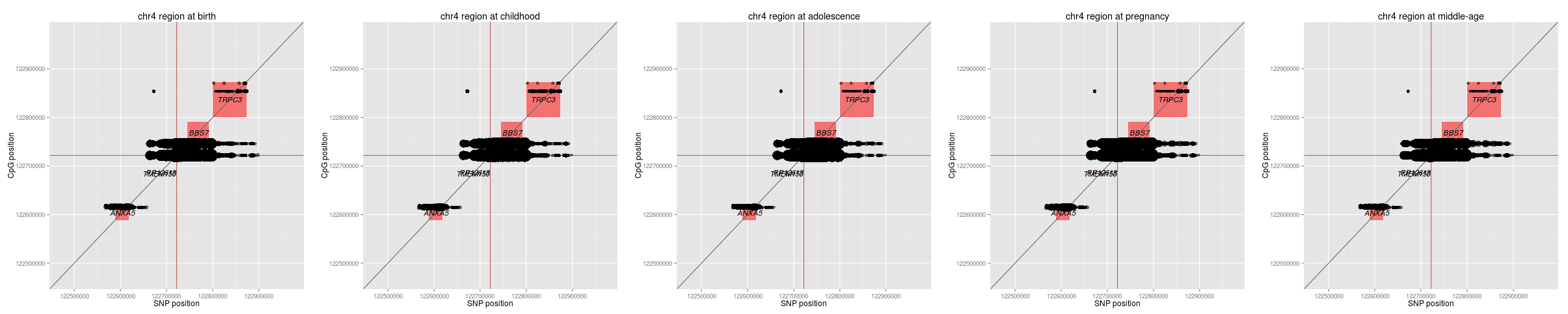


5: cg22863700


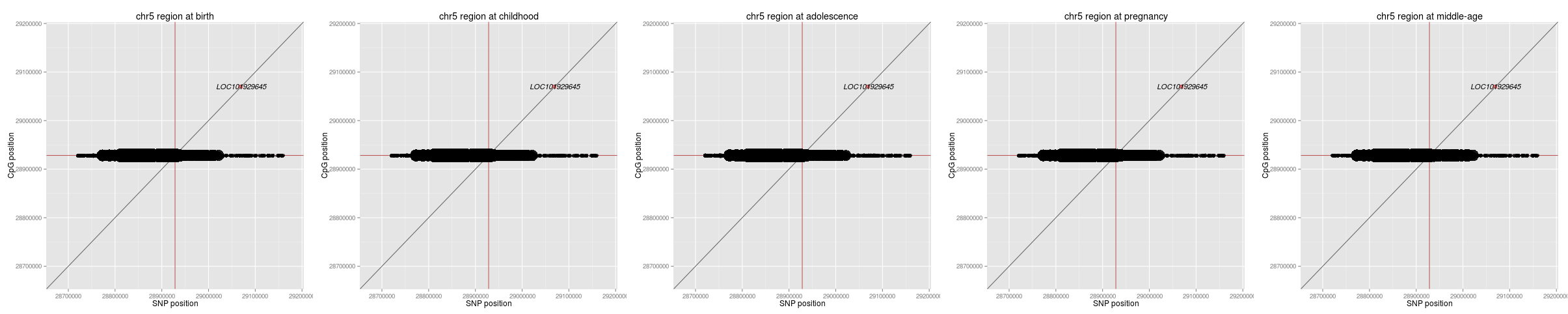


6: cg08109568


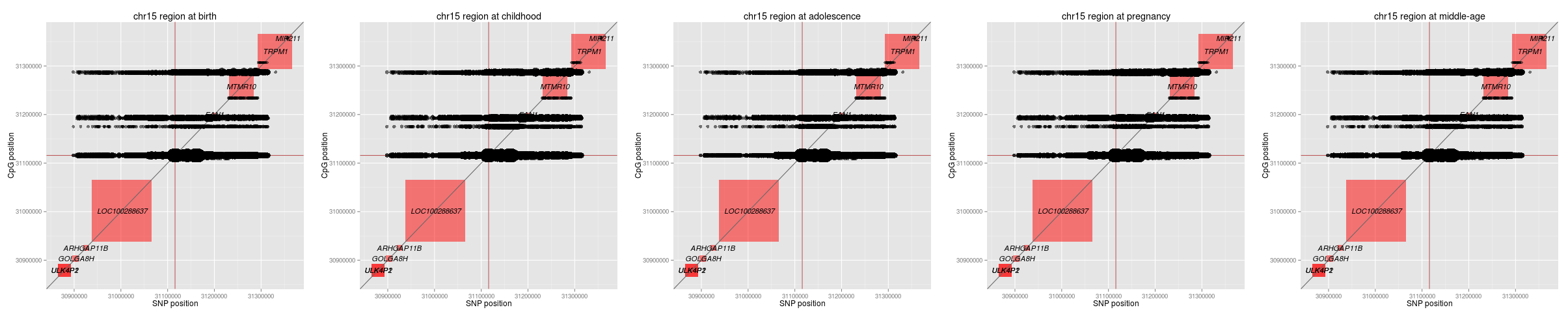


7: cg19078123


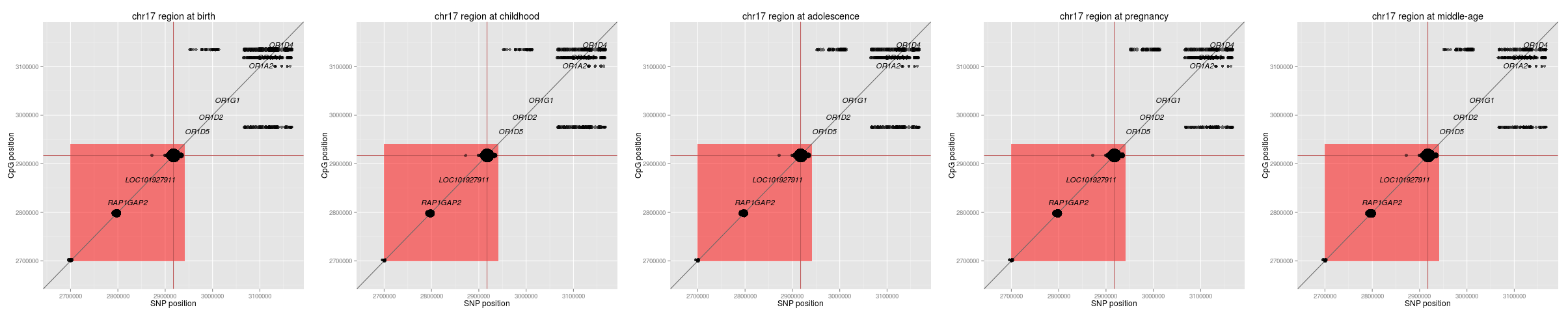


8: cg12489353


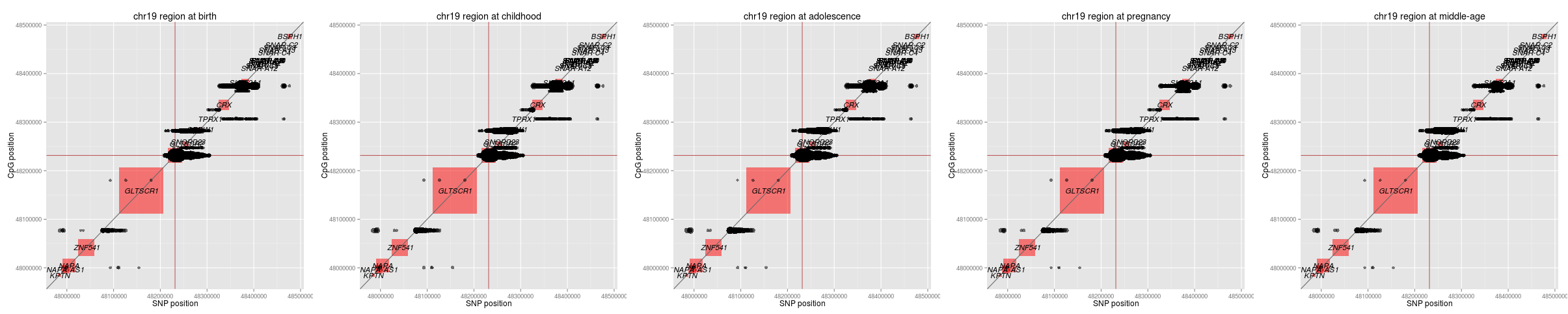


9: cg15059639


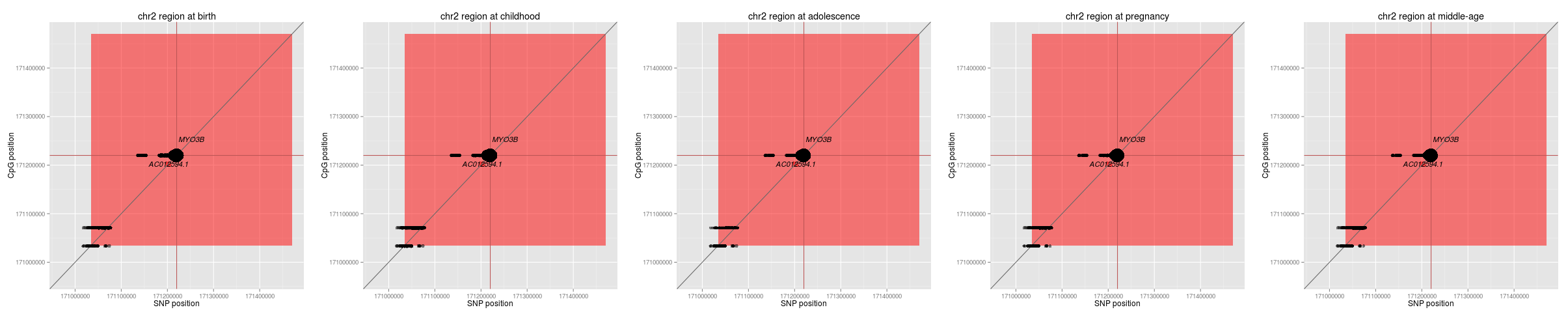


10: cg09660878


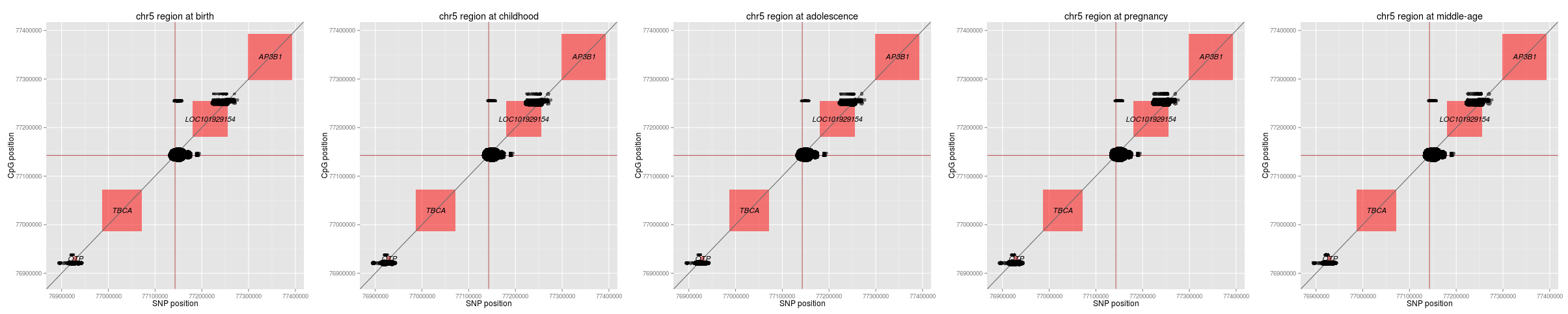


11: cg08509907


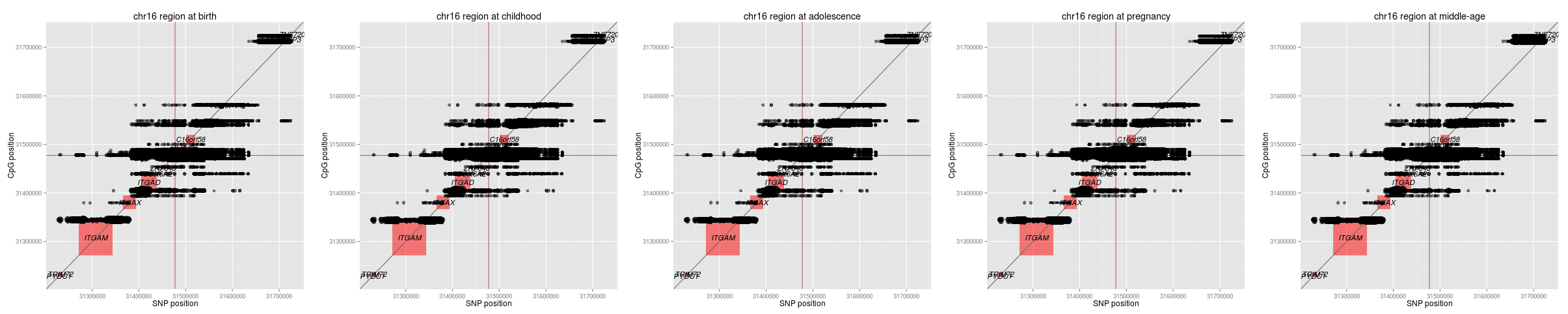


12: cg07629776


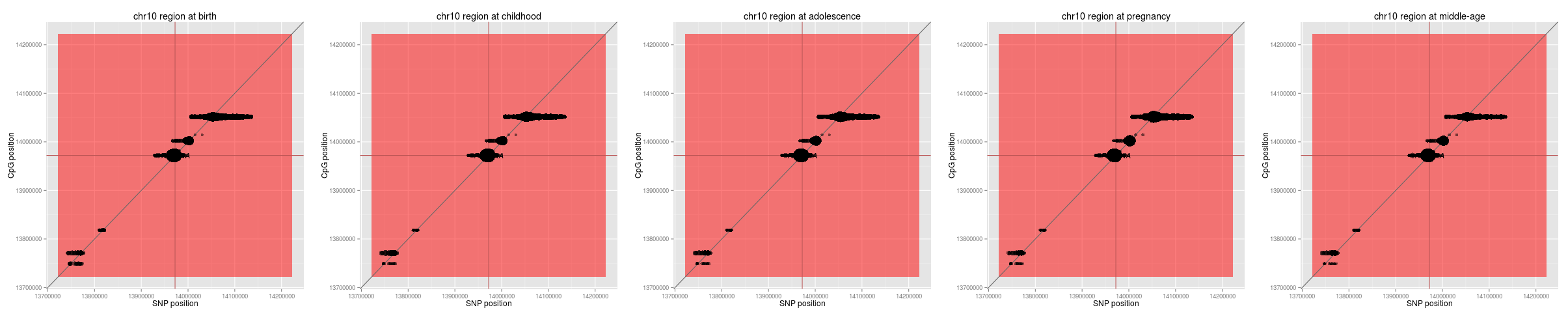


13: cg01821018


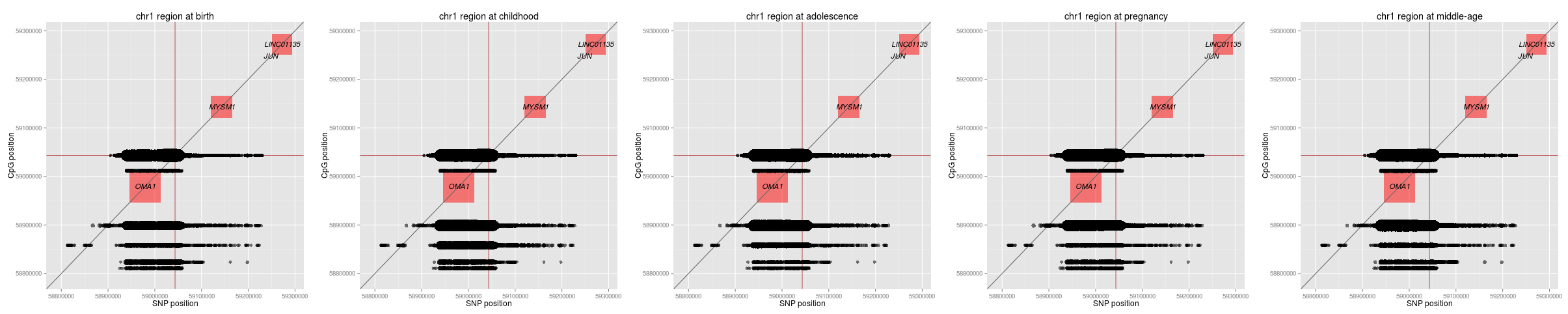


14: cg04863005


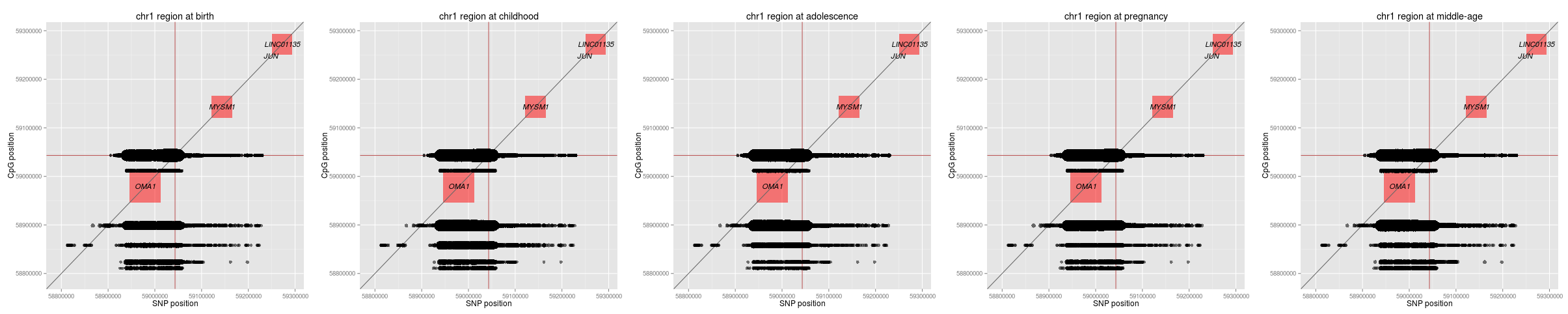


15: cg01624173


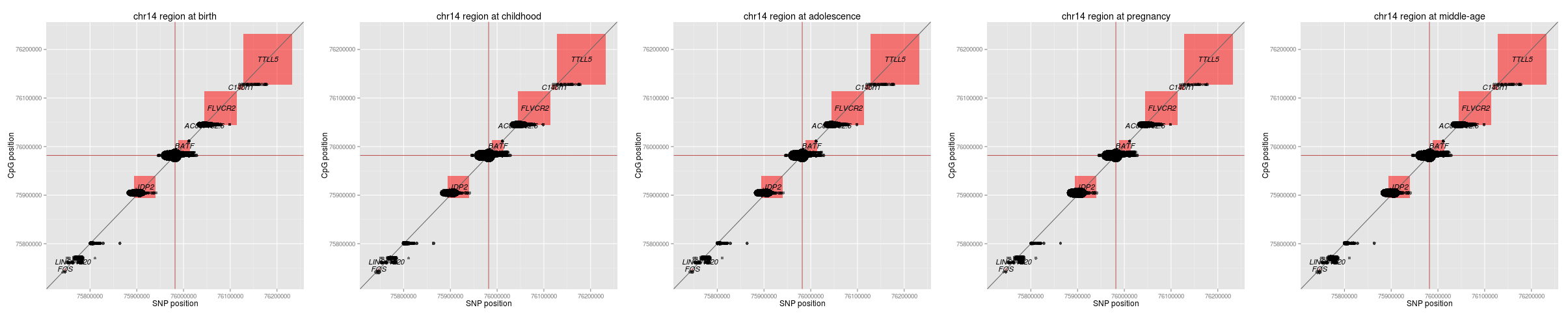


16: cg24851651


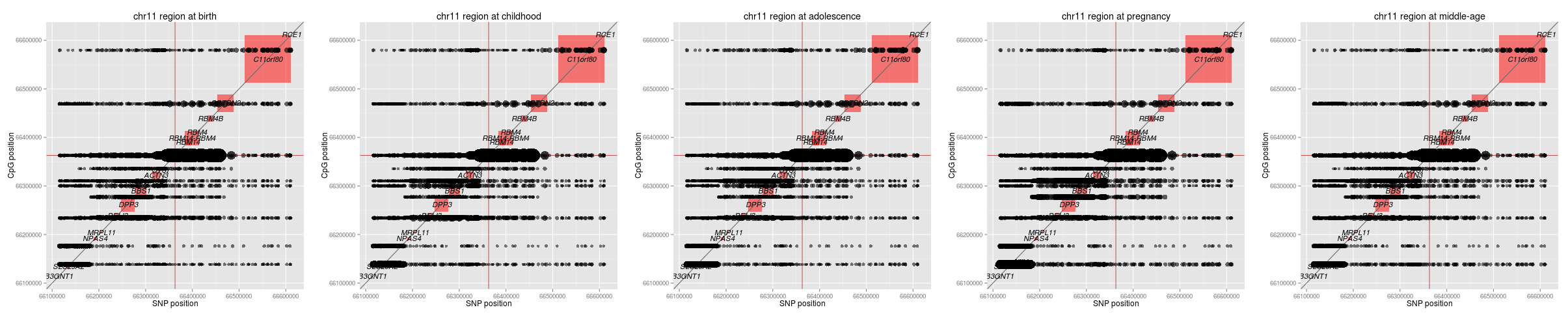


17: cg01062020


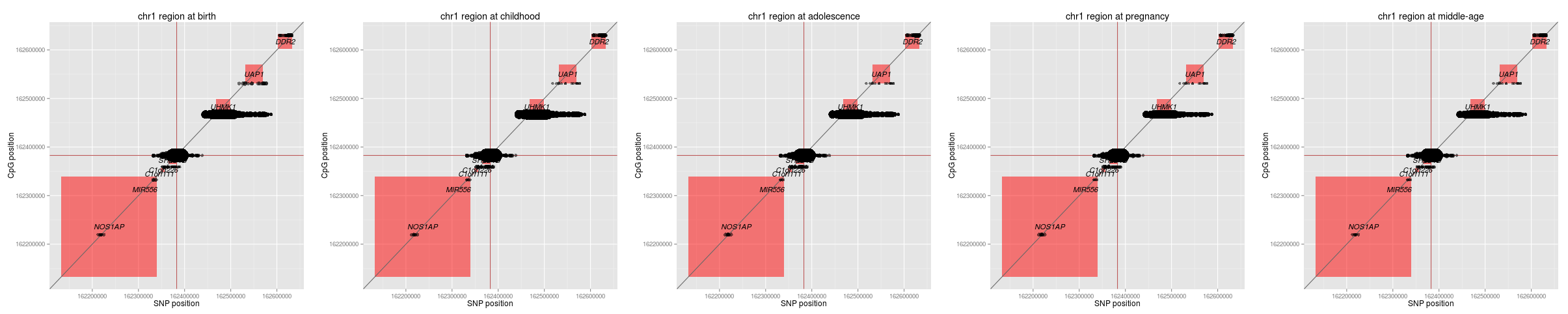


18: cg02210151


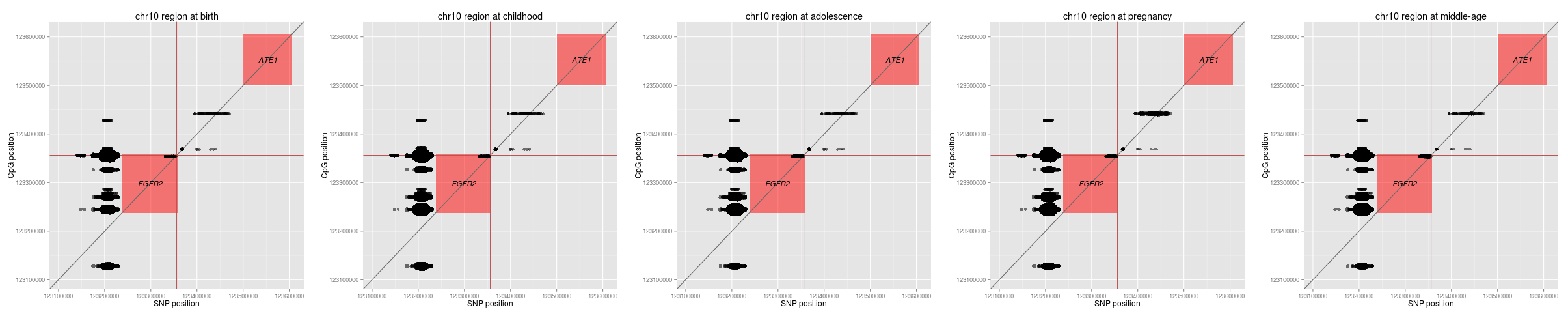


19: cg06791446


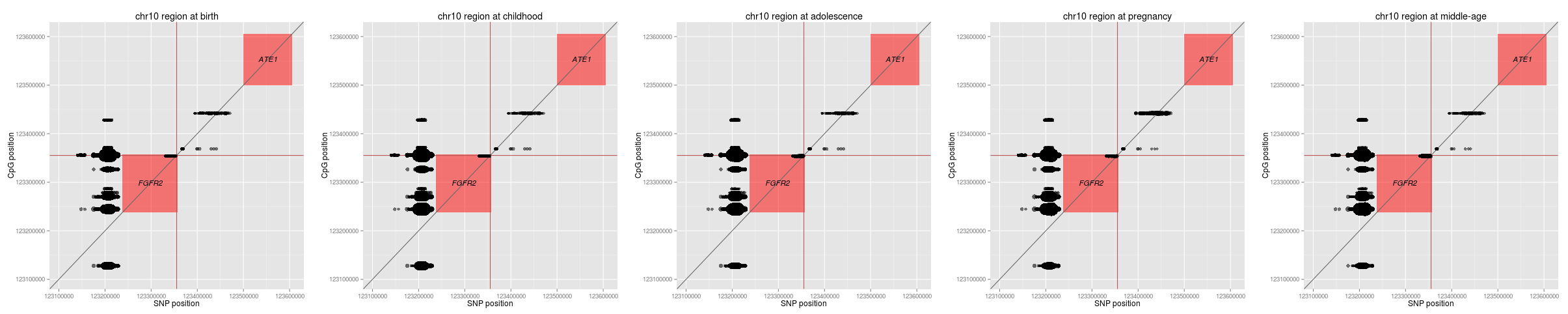


20: cg07044115


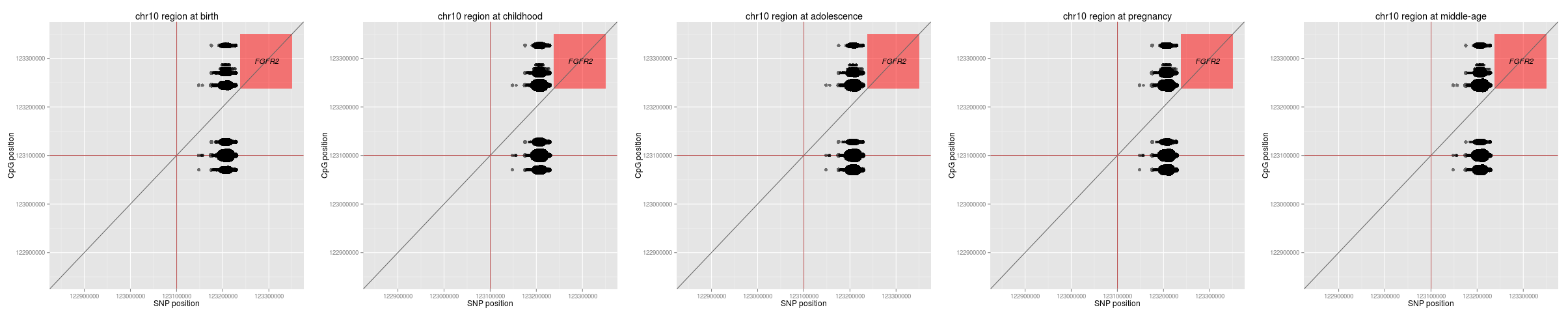


21: cg18566515


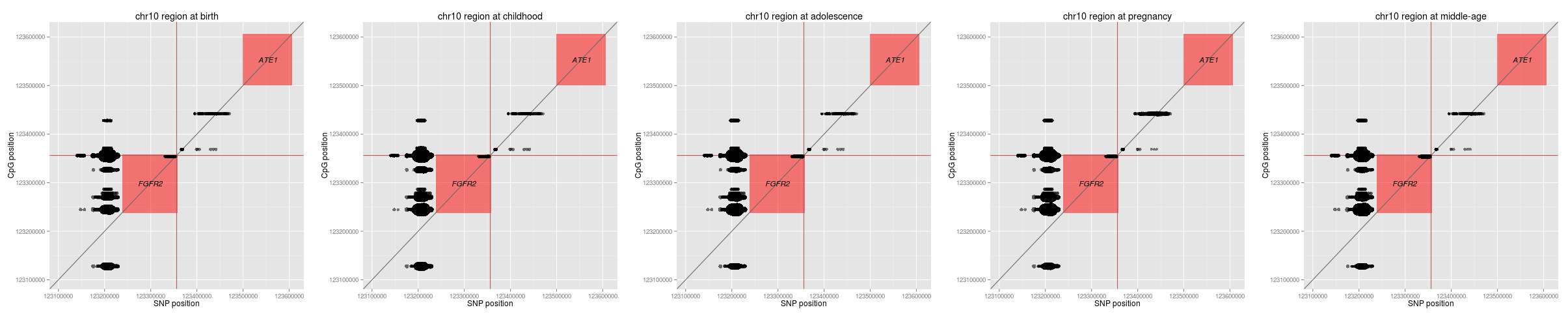


22: cg02754929


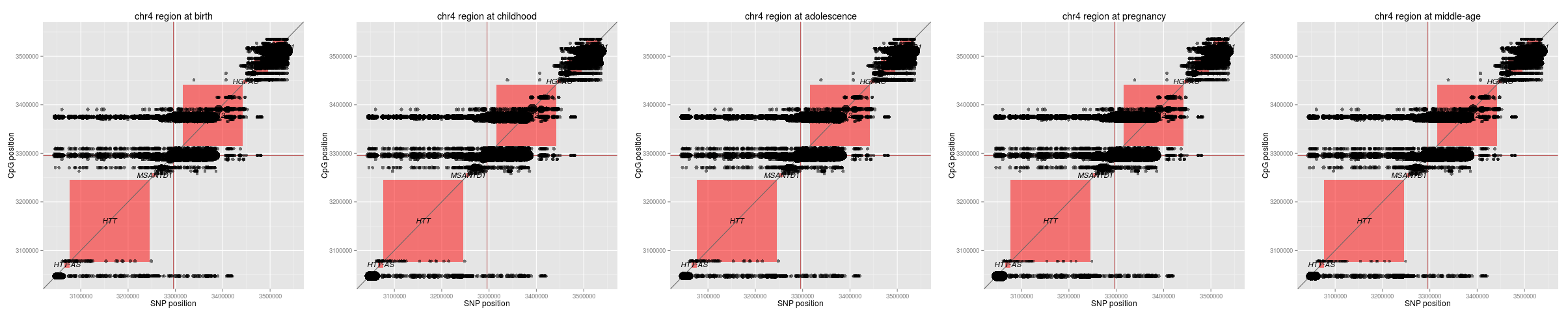


23: cg14829155


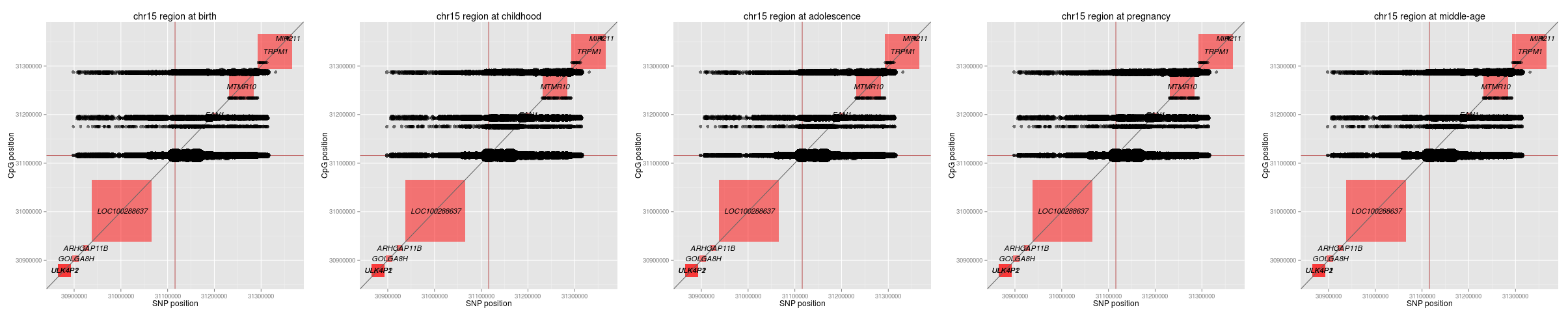


24: cg03807316


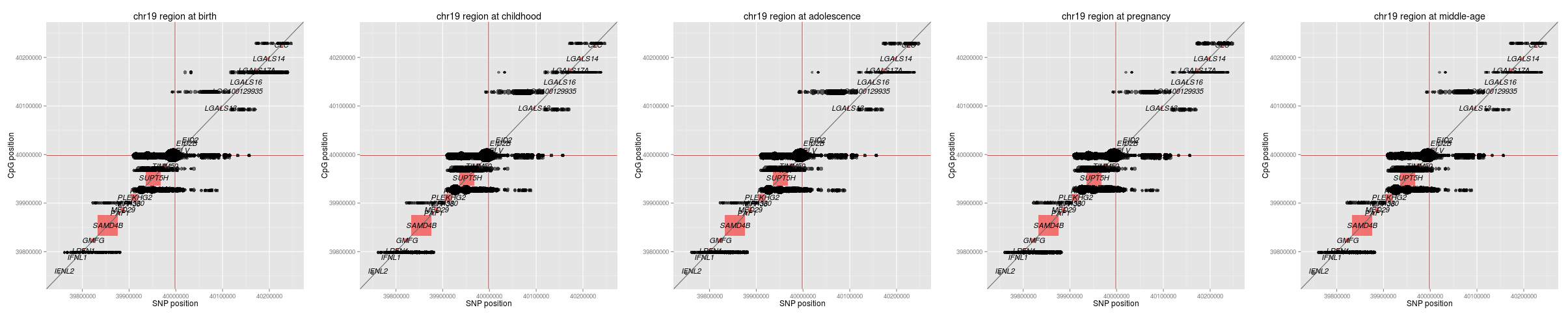


25: cg18477163


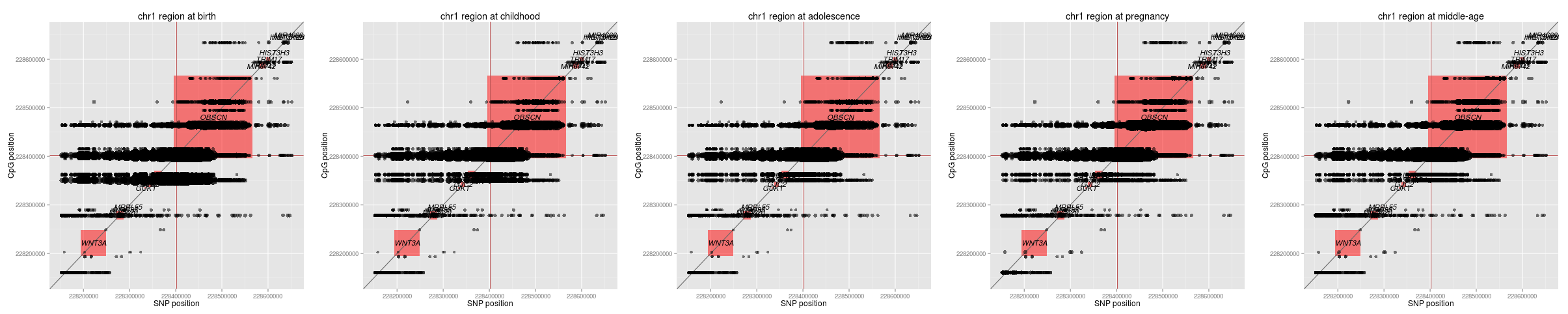


**Figure S12:** Overlap between our cis mQTL and blood cis eQTL reported by the GTEx consortium. For each timepoint: **A** compares the GTEx reported genome-wide significant eQTL with our mQTL at p<1x10^-14^; **B** compares the proportion of most associated GTEx eQTL corresponding to the proportion of our mQTL at p<1x10^-14^. P-value thresholds are shown in parentheses.


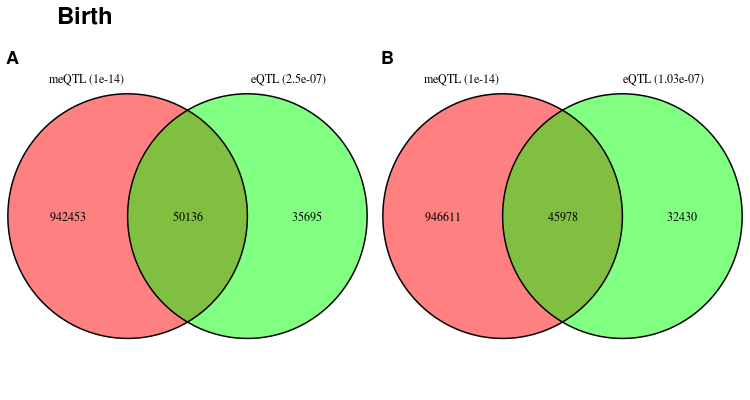

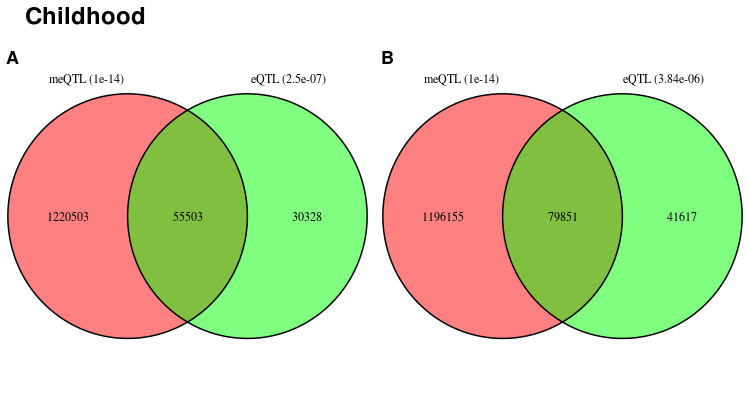


**
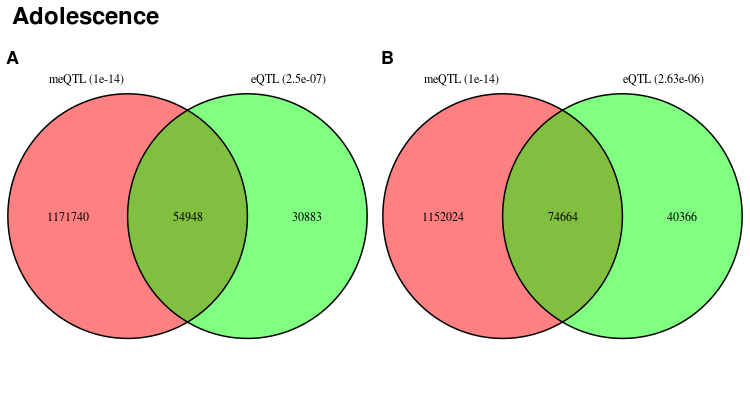
**

**
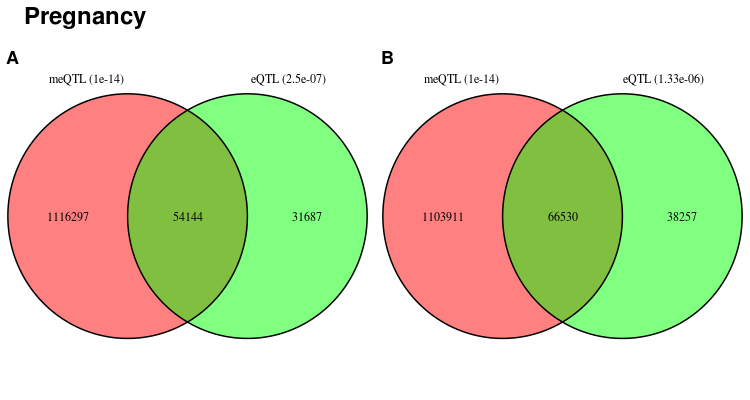

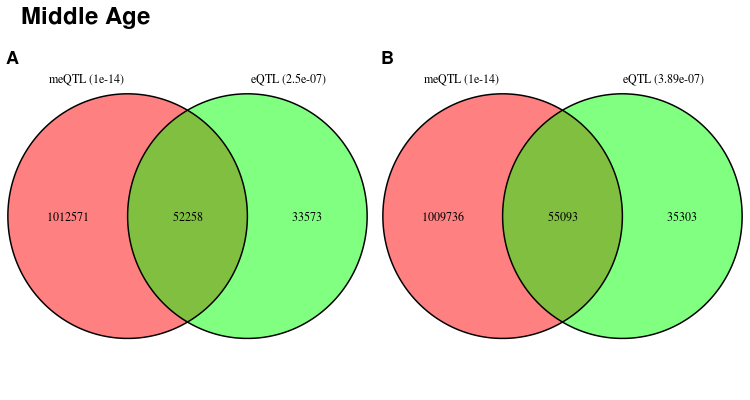
**

**Figure S13:** GO enrichment in CpG sites.


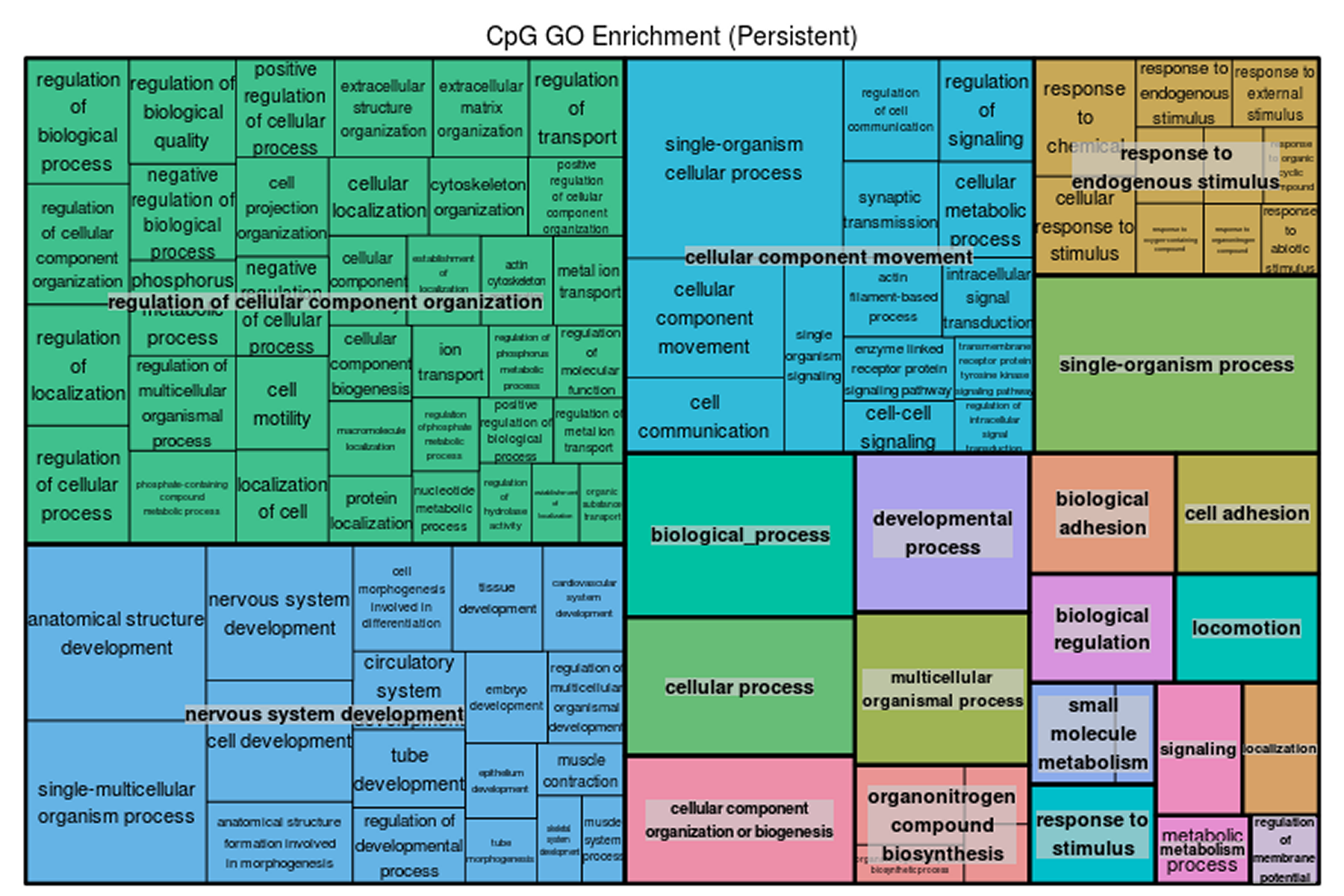


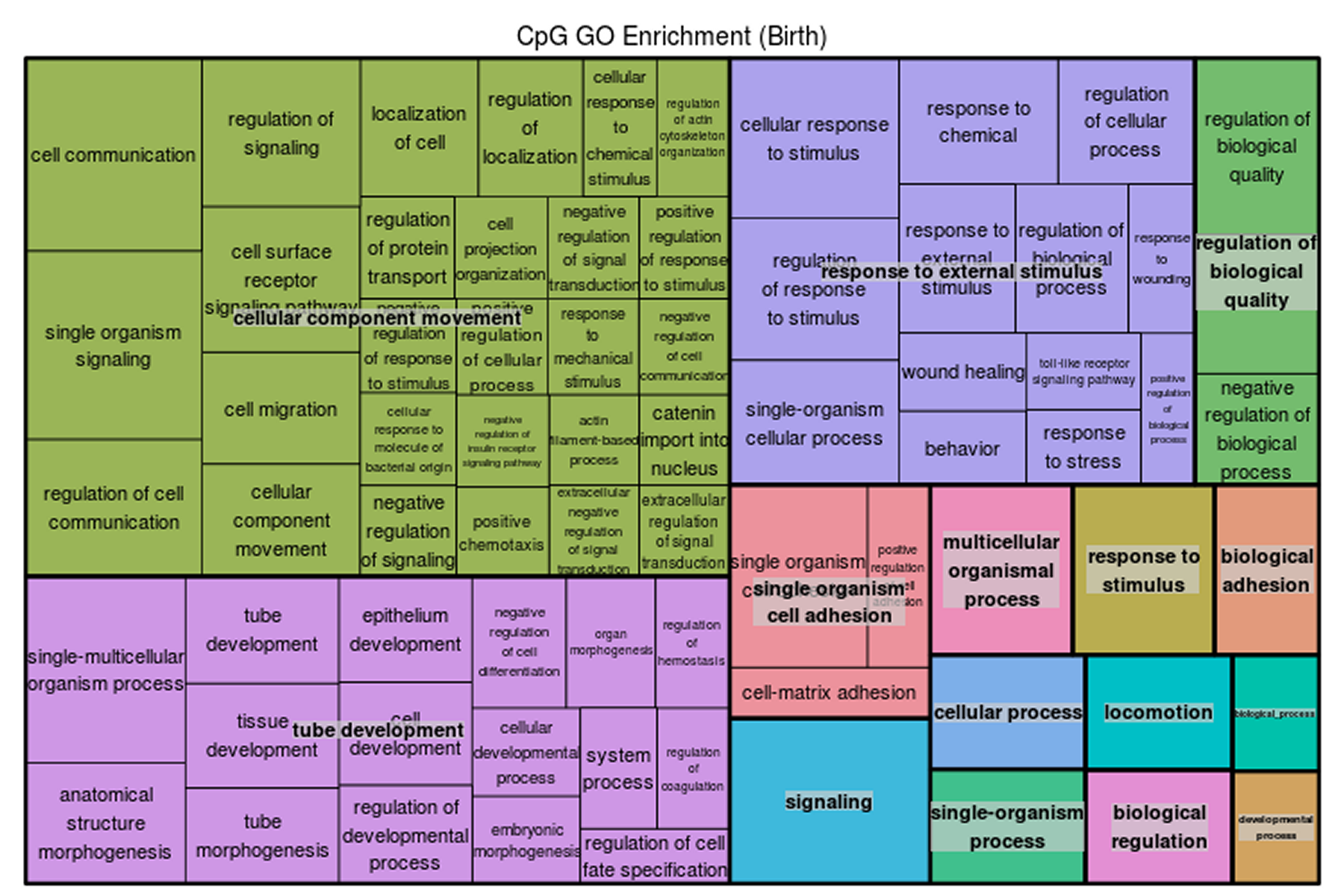


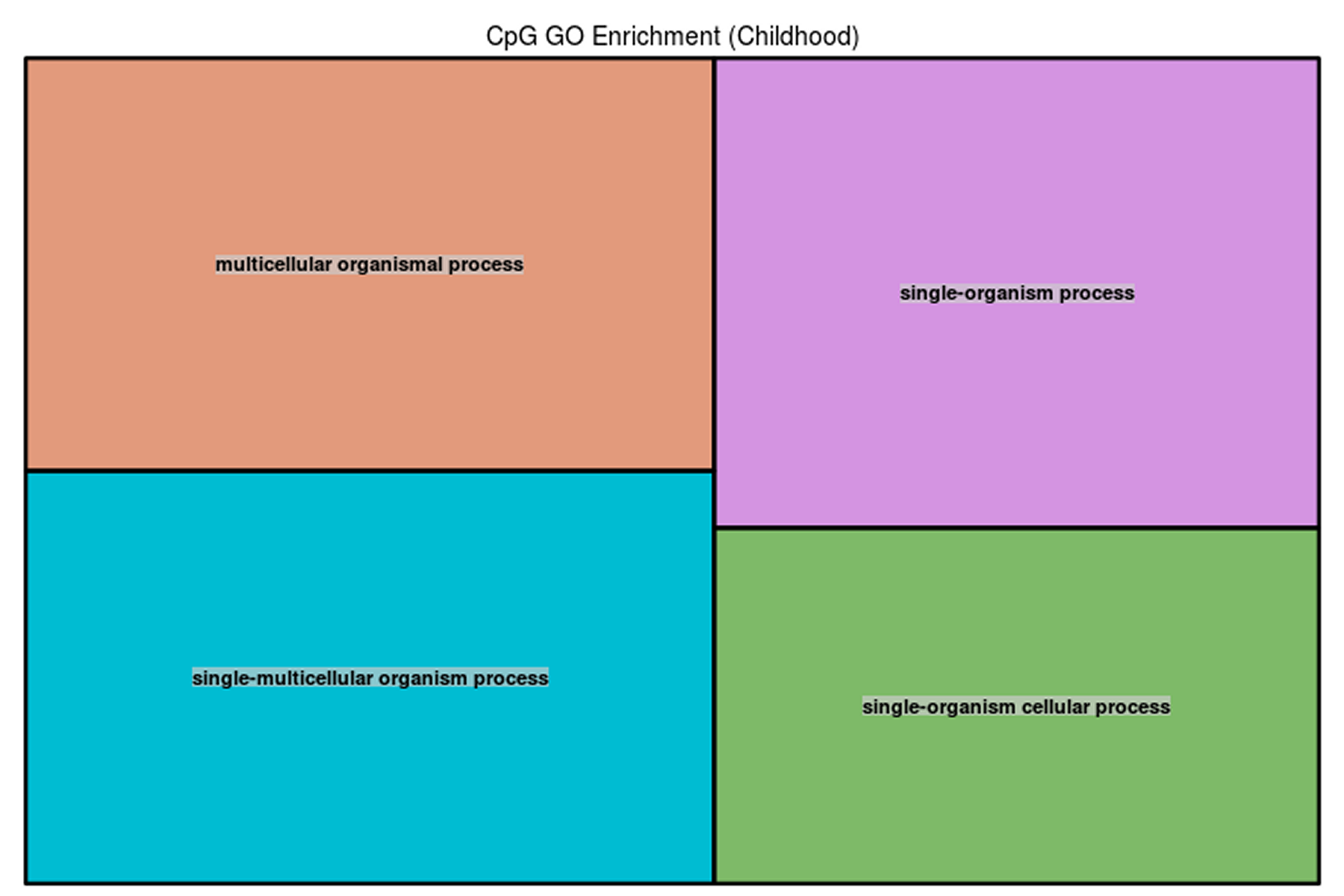


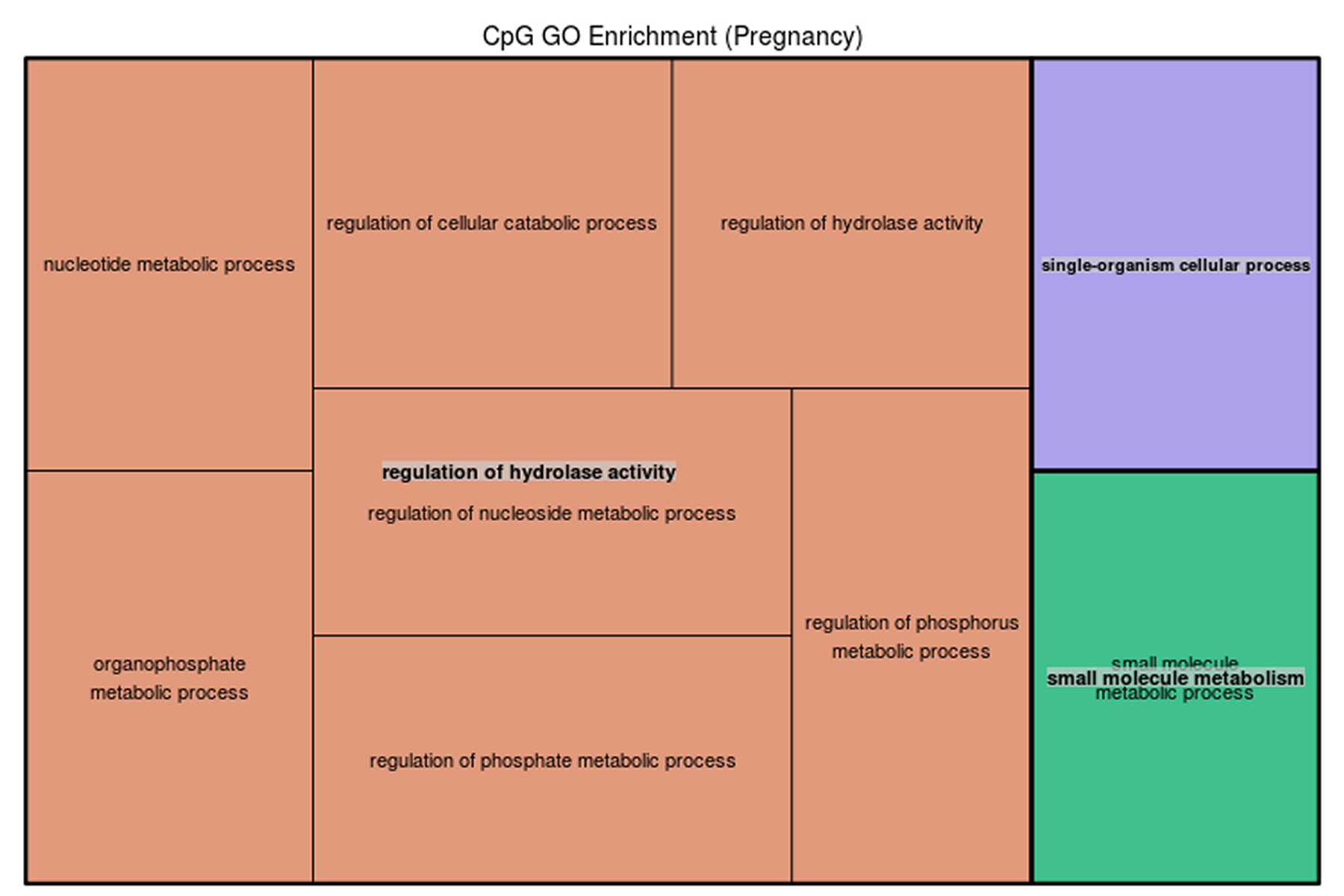


**Figure S14:** GO enrichment in SNP sites.


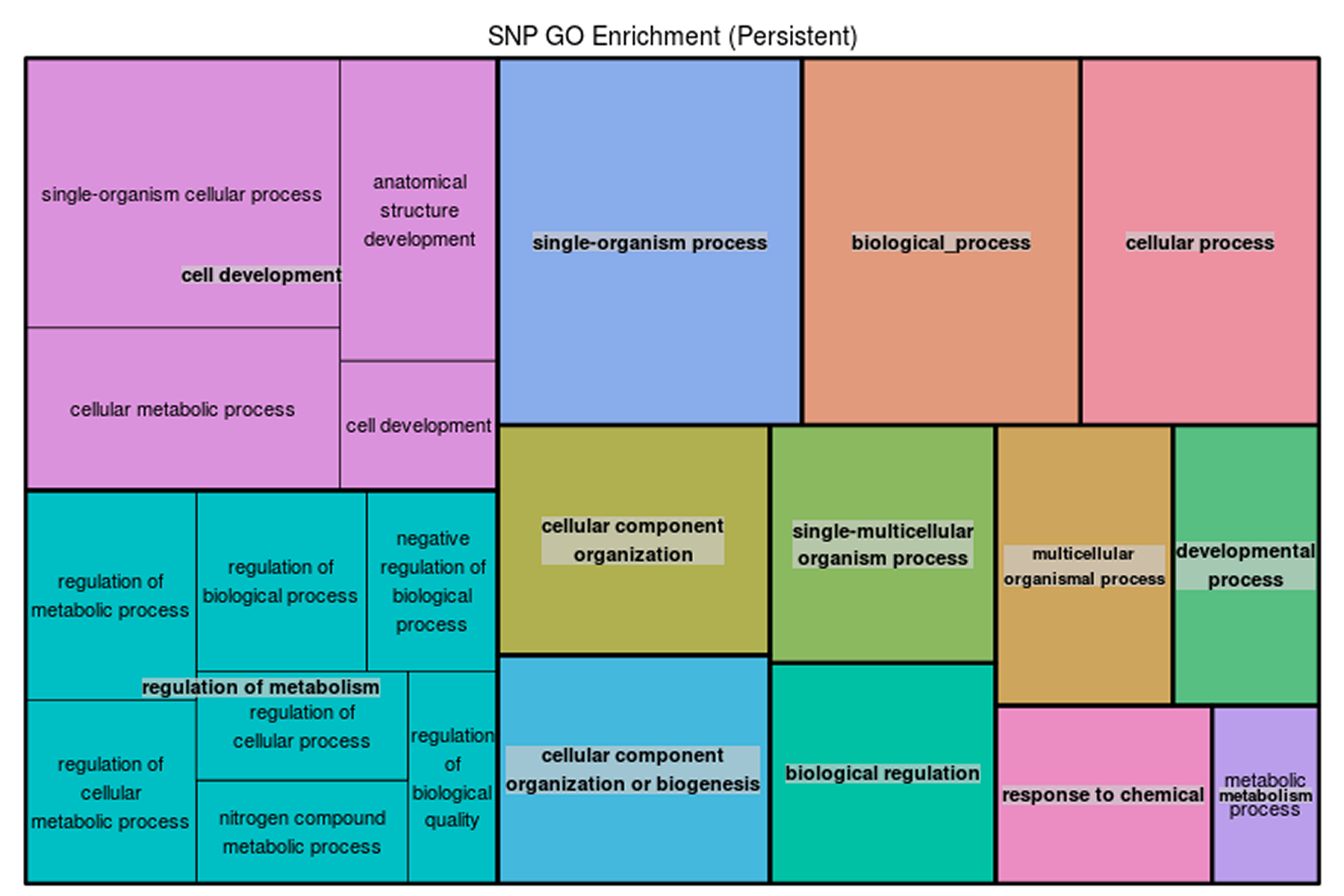


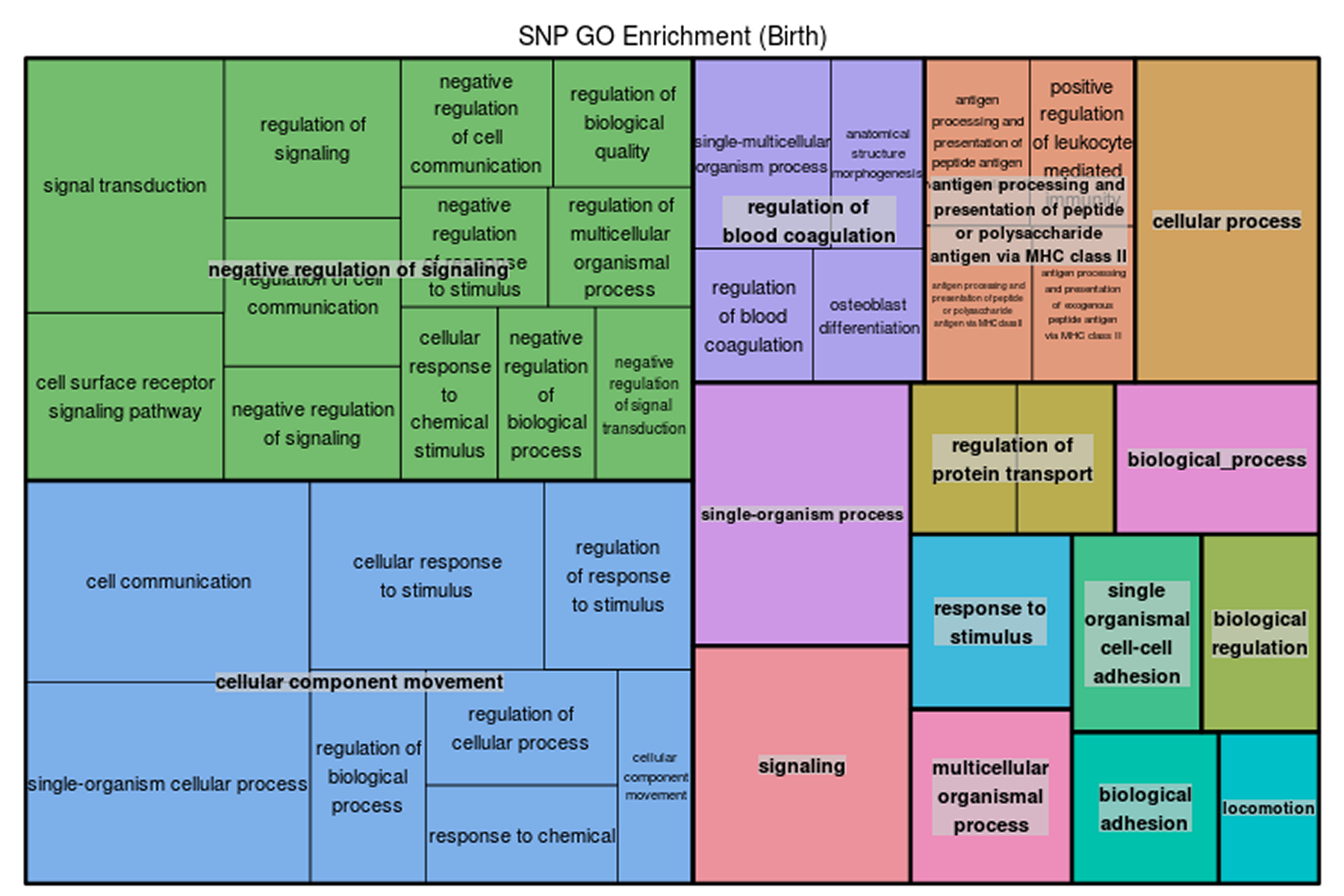


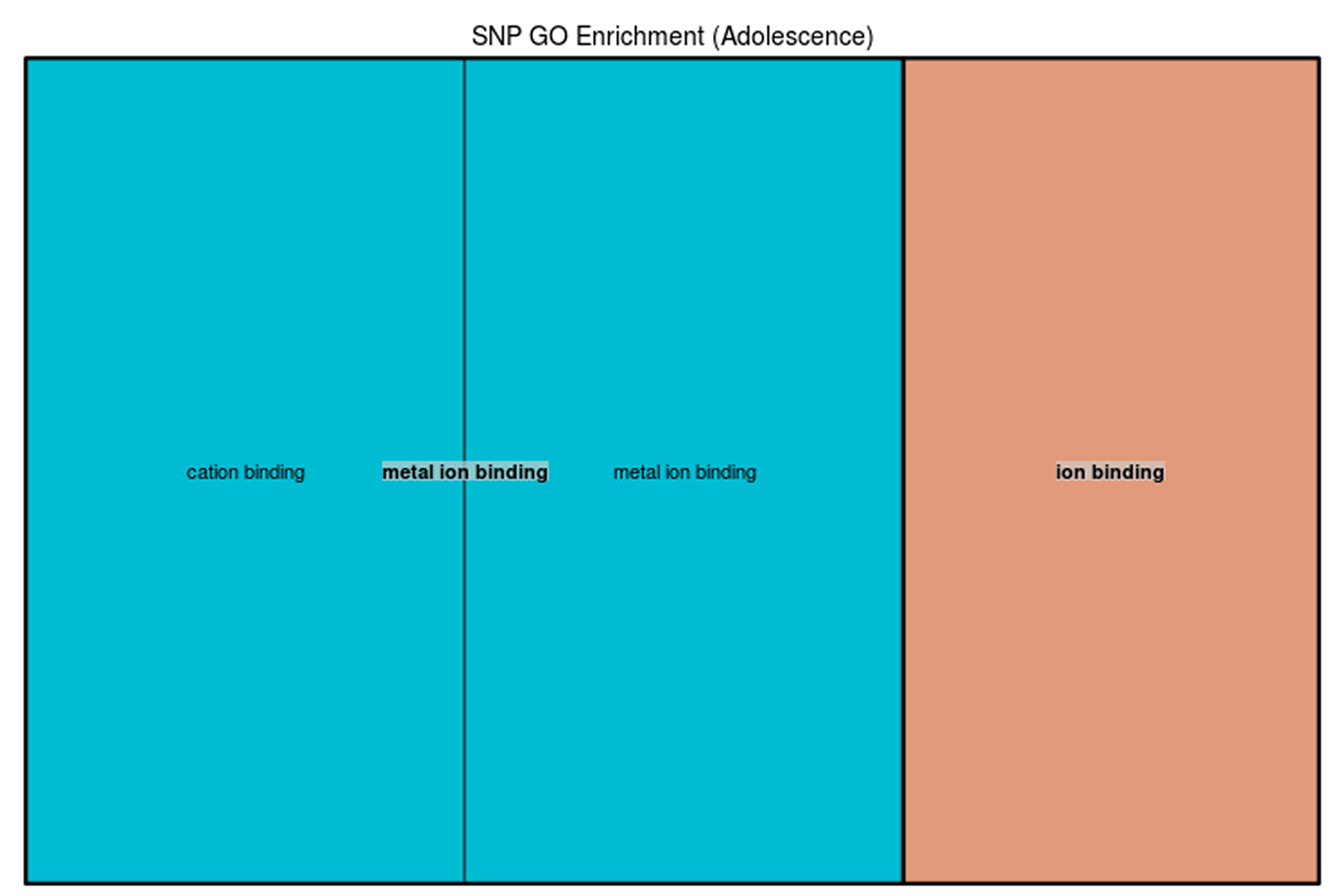


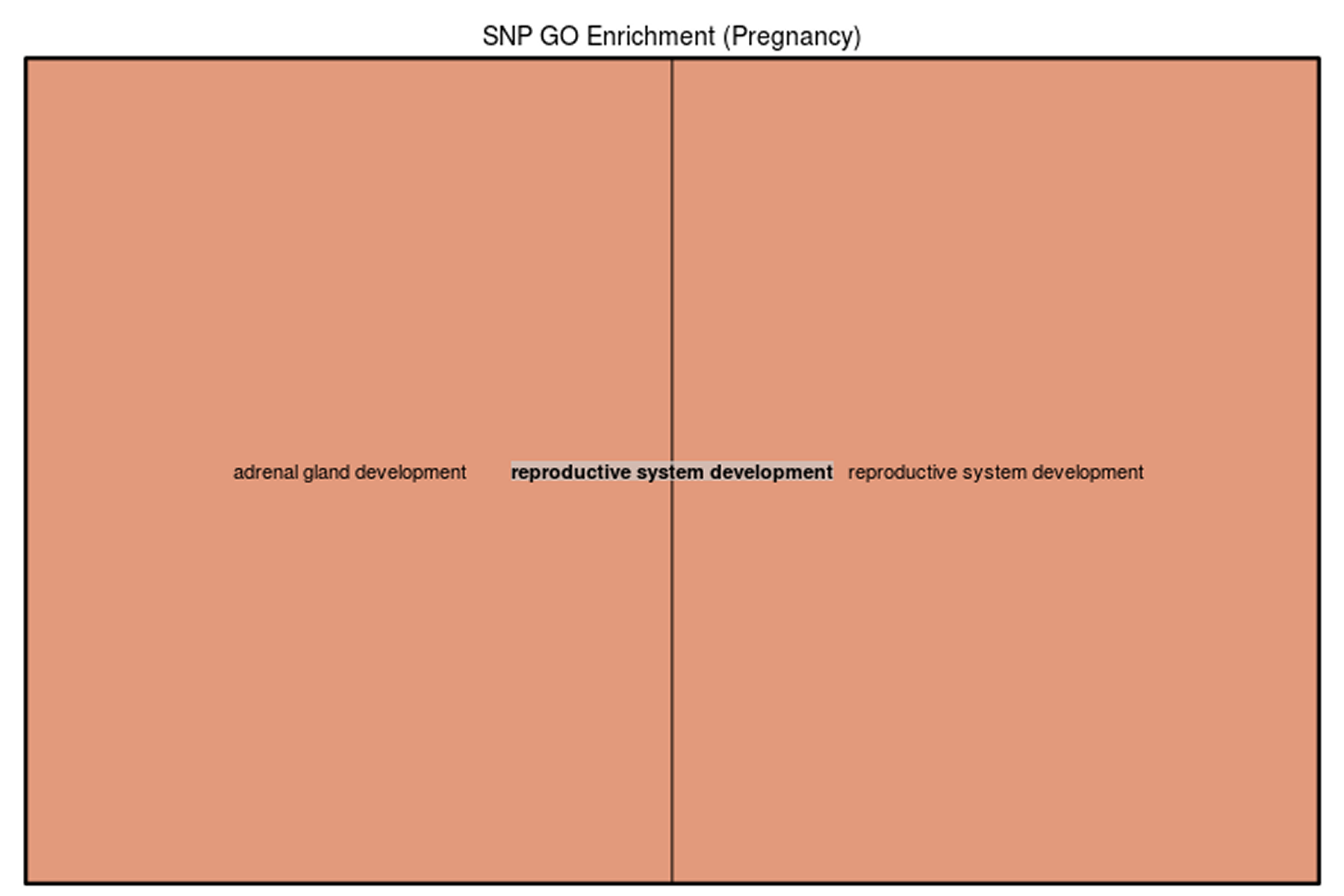


**Figure S15:** GO enrichment in sites reported by us as mQTL and by Westra *et al* as eQTL.


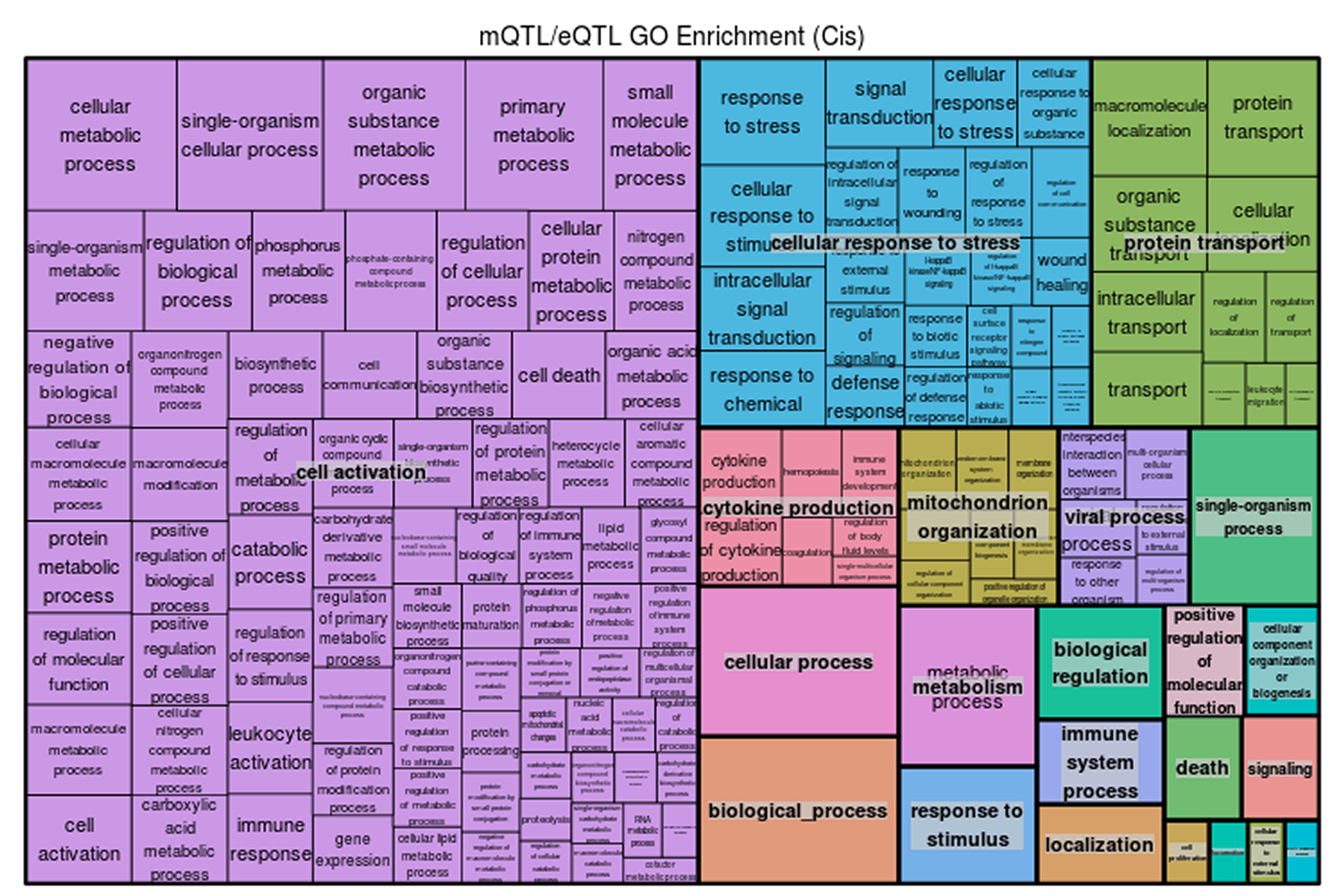


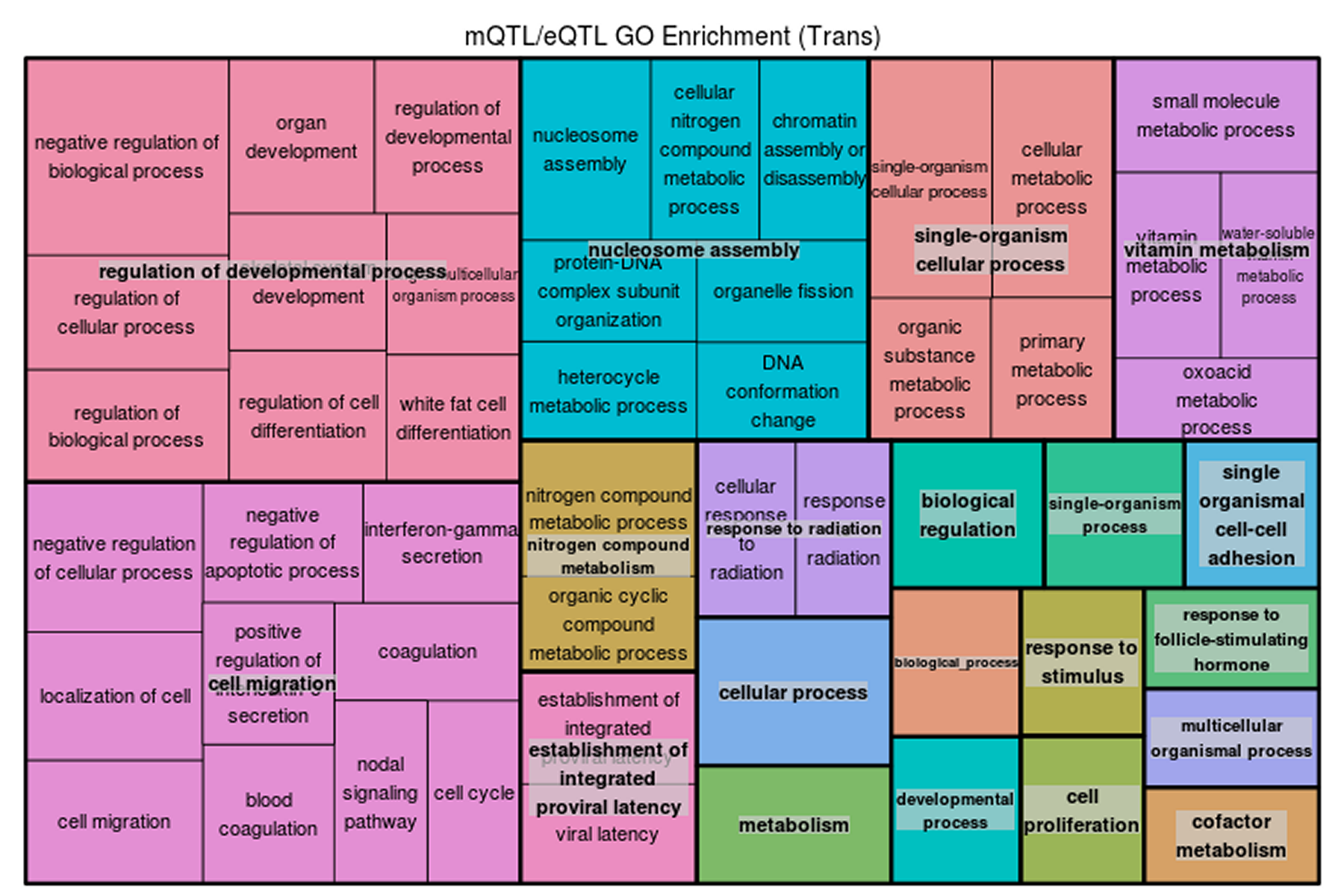


**Figure S16:** Contribution of mQTL identified at each timepoint to variance of (A) BMI, (B) height at different timepoints and (C) WTCCC common diseases bipolar disorder (BD), coronary artery disease (CAD), Crohns disease (CD), hypertension (HT), rheumatoid arthritis (RA), type 1 diabetes (T1D) and type 2 diabetes (T2D). Overall bar height corresponds to the contribution of HapMap3 SNPs to variance in that trait; blue bar height is the component of that attributable to mQTL.

**Figure S17:** The top 20000 most variable methylation probes were extracted for each of the 4 time points for which there were heterogeneous sample types. The first 8 principal components are plotted here, and it shows that there is no clustering of methylation values according to cell type.


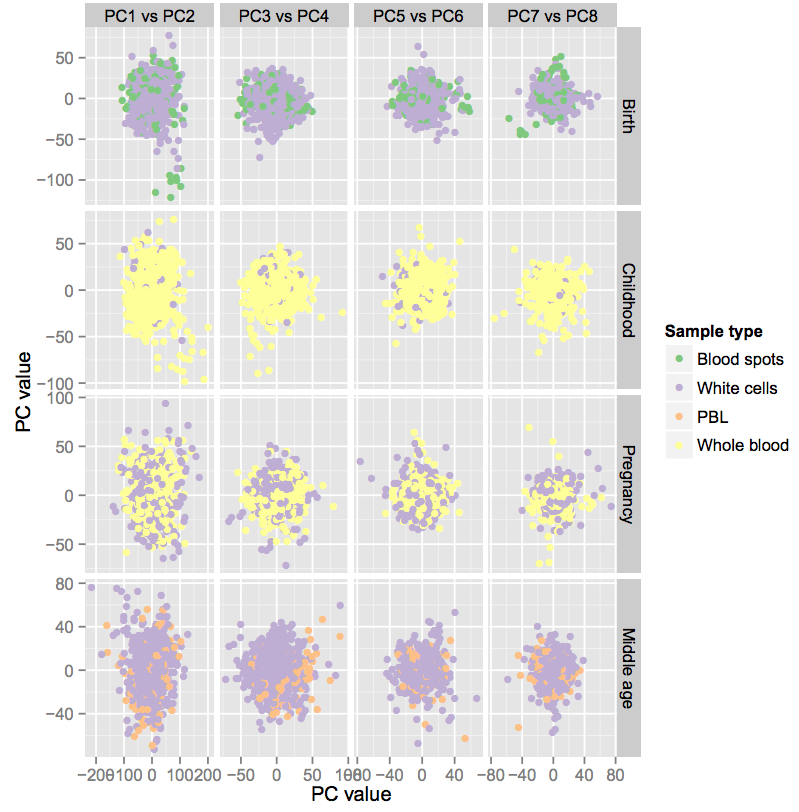


**Figure S18:** Genetic principal components analysis for ARIES participants. ARIES participants (children in left-hand set, mothers in right-hand set) are in dark blue in comparison to HapMap reference data in light blue. Tight clustering indicates little evidence of genetic stratification.


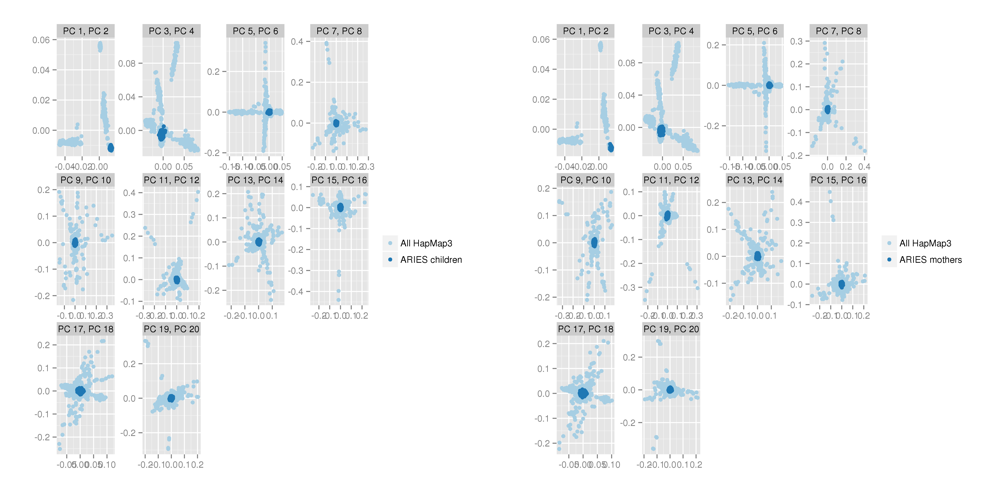


# Supplementary Tables

**Table S1:** Characteristics of participants.

| Time | Proportion Female | Mean Age (SD) |
| --- | --- | --- |
| Birth | 0.49 | NA (all zero) |
| Childhood | 0.50 | 7.49 (0.15) |
| Adolescence | 0.49 | 17.14 (1.01) |
| Pregnancy | NA (all female) | 29.22 (4.41) |
| Middle Age | NA (all female) | 47.45 (4.46) |

**Table S2:** Numbers of samples and variables included in mQTL analyses. eWBC = estimated white blood cell counts. aPCs = ancestry principal components. BCB = bisulphite conversion batch.

| Timepoint | Tissue | # Samples | # SNP | # CpGs | Covariates |
| --- | --- | --- | --- | --- | --- |
| Birth | Cord blood | 771 | 8282911 | 395625 | Sex, eWBC, 10 aPCs, BCB |
| Childhood | Peripheral blood | 834 | 8282911 | 395625 | Sex, age, eWBC, 10 aPCs, BCB |
| Adolescence | Peripheral blood | 837 | 8282911 | 395625 | Sex, age, eWBC, 10 aPCs, BCB |
| Pregnancy | Peripheral blood | 764 | 8282911 | 395625 | Age, eWBC, 10 aPCs, BCB |
| Middle Age | Peripheral blood | 742 | 8282911 | 395625 | Age, eWBC, 10 aPCs, BCB |

**Table S3:** Total numbers of cis and trans mQTL associations at each time point at p<1x10^-14^. Original discovery was performed using Matrix eQTL, and replicated across all time points followed by joint analysis with GCTA to determine number of independent associations and loci. Counts are divided into cis and trans mQTL at each time point. Total refers to the total number of SNP-CpG associations; UniqSNP is the total number of SNPs associated with 1 or more CpGs; total number of CpGs associated with 1 or more SNPs.

|  | Birth |  | Childhood |  | Adolescence |  | Pregnancy |  | Middle Age |  |
| --- | --- | --- | --- | --- | --- | --- | --- | --- | --- | --- |
|  | Cis | Trans | Cis | Trans | Cis | Trans | Cis | Trans | Cis | Trans |
| Matrix eQTL |  |  |  |  |  |  |  |  |  |  |
| Total | 2449164 | 221416 | 3609643 | 319638 | 3447280 | 295930 | 3177648 | 272654 | 2786186 | 241582 |
| UniqSNP | 1142663 | 148218 | 1487090 | 203522 | 1430443 | 189114 | 1360455 | 177574 | 1231136 | 160062 |
| UniqCpG | 25795 | 2042 | 35054 | 2739 | 33228 | 2544 | 31651 | 2453 | 28938 | 2169 |
| PLINK |  |  |  |  |  |  |  |  |  |  |
| Total | 2449200 | 221419 | 3609657 | 319639 | 3447304 | 295933 | 3177681 | 272655 | 2786225 | 241591 |
| UniqSNP | 1142673 | 148218 | 1487095 | 203522 | 1430447 | 189115 | 1360461 | 177575 | 1231146 | 160071 |
| UniqCpG | 25795 | 2042 | 35054 | 2739 | 33228 | 2544 | 31651 | 2453 | 28938 | 2169 |
| GCTA |  |  |  |  |  |  |  |  |  |  |
| Total | 26967 | 1979 | 37175 | 2658 | 35334 | 2442 | 33492 | 2394 | 30506 | 2144 |
| UniqSNP | 22286 | 1735 | 29909 | 2306 | 28643 | 2131 | 27239 | 2069 | 24842 | 1889 |
| UniqCpG | 25583 | 1905 | 34364 | 2520 | 32736 | 2320 | 31207 | 2280 | 28733 | 2045 |
